# Analysis of the *TCP* genes expressed in the inflorescence of the orchid *Orchis italica*

**DOI:** 10.1038/srep16265

**Published:** 2015-11-04

**Authors:** Sofia De Paolo, Luciano Gaudio, Serena Aceto

**Affiliations:** 1 Department of Biology, University of Naples Federico II, Napoli, Italy

## Abstract

TCP proteins are plant-specific transcription factors involved in many different processes. Because of their involvement in a large number of developmental pathways, their roles have been investigated in various plant species. However, there are almost no studies of this transcription factor family in orchids. Based on the available transcriptome of the inflorescence of the orchid *Orchis italica*, in the present study we identified 12 transcripts encoding TCP proteins. The phylogenetic analysis showed that they belong to different TCP classes (I and II) and groups (PCF, CIN and CYC/TB1), and that they display a number of conserved motifs when compared with the TCPs of *Arabidopsis* and *Oryza*. The presence of a specific cleavage site for the microRNA miRNA319, an important post-transcriptional regulator of several *TCP* genes in other species, was demonstrated for one transcript of *O. italica*, and the analysis of the expression pattern of the *TCP* transcripts in different inflorescence organs and in leaf tissue suggests that some *TCP* transcripts of *O. italica* exert their role only in specific tissues, while others may play multiple roles in different tissues. In addition, the evolutionary analysis showed a general purifying selection acting on the coding region of these transcripts.

The *TCP* genes encode plant-specific transcription factors that regulate plant growth and development by controlling cell proliferation. Initially identified in angiosperms, members of the TCP family have also been found in green algae, mosses, ferns, lycophytes and gymnosperms, showing that their origin predates the emergence of land plants^1^. The members of this large family of proteins share the TCP domain, a non-canonical ∼60 amino acid basic helix-loop-helix (bHLH) motif involved in DNA binding, nuclear localization and protein-protein interactions^2^,^3^. The acronym TCP derives from the name of the proteins where this domain was initially described: TEOSINTE BRANCHED1 (TB1) from *Zea mays*^4^, CYCLOIDEA (CYC) from *Antirrhinum majus*^5^ and PROLIFERATING CELL FACTORS 1 and 2 (PCF1 and PCF2) from *Oryza sativa*^6^.

Based on the sequence differences in the TCP domain, the members of the TCP family can be classified into two subfamilies, class I (or PCF or TCP-P) and class II (or TCP-C)^1^,^3^,^7^. The main difference between these two groups is the presence of an additional short amino acid stretch (four residues) in the basic domain of the class II proteins. In addition, the consensus binding site on their DNA targets is different, even if overlapping: GGNCCCAC for class I and G(T/C)GGNCCC for class II proteins^7^,^8^. Outside the TCP domain, some members of the class II subfamily present an arginine-rich motif of 18–20 amino acids called the R domain, whose function is unknown^2^. The members of the class II subfamily are quite heterogeneous and can be further classified into two sub-clades: the CYC/TB1-like group, also known as the ECE group based on the presence of a glutamic acid-cysteine-glutamic acid stretch^9^, and the CIN-like group^3^. Both class I and class II members are present in some green algae, making it difficult to establish which TCP subfamily is more ancient. In contrast, the apparent absence of *CYC/TB1*-like genes (class II) in mosses and lycophytes supports the hypothesis that the *CIN*-like genes are more ancestral^10^.

The role of class I *TCP* genes during plant development is mainly to promote cell division, growth and differentiation, and most of them act in a redundant manner^6^,^11^. In contrast, class II genes are generally involved in the inhibition of cell growth and proliferation^3^, and their expression is strictly controlled at various transcriptional and post-transcriptional levels. For example, some angiosperm members of the CIN-like clade involved in leaf and flower morphogenesis are targeted by the microRNA miR319^12^,^13^,^14^,^15^,^16^. Recently, the role of TCP proteins has been demonstrated in plant immunity responses, through the interaction with pathogenic effectors^17^, and in mitochondrial metabolism, through the binding to the *cis*-acting regulatory element TGGGGCY^18^. Another relevant role of the *TCP* genes, in particular the members of the CYC/TB1-like clade, is the establishment of the floral monosymmetry through their interactions with MYB transcription factors and cell cycle proteins^19^,^20^,^21^. Zygomorphic flowers have evolved independently multiple times during the angiosperm evolution. The involvement of *CYC/TB1*-like genes in the evolution and maintenance of bilateral symmetry in dicots has been extensively analyzed^5^,^22^,^23^,^24^,^25^,^26^,^27^,^28^, whereas only a few studies are available for monocots^29^,^30^,^31^,^32^. Other members of the *CYC/TB1*-like genes, e.g. *TB1* of *Zea mays* and its orthologs in dicots *BRC1*, are involved in the control of lateral branches^33^,^34^. Among monocots, the family Orchidaceae is characterized by highly specialized zygomorphic flowers, and to date, *CYC/TB1*-like genes have not been identified in orchids^35^.

The application of the next-generation sequencing techniques is producing an increasing amount of data that opens new opportunities to study the *TCP* genes at the genomic and transcriptomic level^36^,^37^,^38^,^39^ in non-model species, including orchids^40^,^41^,^42^. The family Orchidaceae is one of the most species-rich plant families including species with highly specialized morphological and physiological characteristics. *Orchis italica* (sub-family Orchidoideae, tribe Orchidinae) is an Eurasian orchid species. Its inflorescence is oval and dense, with numerous pink flowers that exhibit the typical organization of the orchid floral organs^43^. In brief, there are three sepals (outer tepals) and three petals distinguished in two inner lateral tepals and one inner median tepal (lip or labellum). The column is a fusion of male and female reproductive tissues, at the top of which are located the pollen grains. The maturation of the ovary, placed at the base of the column, is triggered by pollination^44^,^45^.

In the present paper, we report the transcriptome-wide identification of the *TCP* genes expressed in the inflorescence of *Orchis italica* based on the inflorescence transcriptome^46^ and miRNAome^47^ available for this wild Mediterranean species. We examined the expression patterns of the identified *TCP* transcripts and of the microRNA miR319 in different tissues and performed a comparative analysis to identify conserved motifs among the TCP proteins of *O. italica,* other orchid species and the model species, *Arabidopsis thaliana* and *Oryza sativa*.

## Results and Discussion

### Transcripts encoding TCP proteins in the inflorescence of *O. italica*

To identify transcripts encoding TCP proteins, the inflorescence transcriptome of *O. italica*^46^ was screened through a standalone TBLASTN search using as query the sequences of the TCP DNA-binding domain of *A. thaliana* and *O. sativa*. Eleven not-redundant transcripts (ranging from 850 to 2788 bp) present in the inflorescence transcriptome of *O. italica* contain a region encoding the TCP domain (Supplementary Data S1 and Figure S1) flanked downstream by at least 500 bp of additional sequence. This indicates that at least eleven distinct *TCP* genes are expressed in the inflorescence of *O. italica*, increasing the small number of known *TCP* genes of orchids (five in *Phalaenopsis hybrid cultivar* and one in *Dendrobium hybrid cultivar*)^35^. Surprisingly, a first phylogenetic analysis of these virtually translated sequences of *O. italica* and the TCP proteins of *Arabidopsis thaliana* and *Oryza sativa* revealed that none of the *TCP* transcripts present in the inflorescence transcriptome of *O. italica* belong to the class II CYC/TB1-like group, whose members are involved in the establishment of bilateral symmetry in numerous plant species^26^,^28^,^48^,^49^,^50^,^51^. The recent release of the assembled genome of the orchid species *Phalaenopsis equestris*^42^ provided for the first time the opportunity to check for the presence of *TCP* genes belonging to the class II CYC/TB1-like group within the genome of an orchid species. Using the *TCP* transcripts of *O. italica* as queries, standalone BLASTN and BLASTX searches of the assembled genome of *P. equestris* were conducted. In addition, the annotated sequences of the genome of *P. equestris* were checked to verify the presence of additional TCP genes. Excluding three sequences with an incomplete TCP domain, in the genome of *P. equestris* there are 17 TCP-encoding genes and among them three belong to the CYC/TB1-like group (Supplementary Table S1). To check for the presence of CYC/TB1-like sequences in *O. italica*, degenerate primers based on the CYC/TB1-like sequences of *P. equestris* were used to amplify the genomic DNA of *O. italica*. The single fragment of 378 bp obtained from this PCR amplification (named *OitaTB1* and deposited in GenBank with the accession number KR858306) encodes the region from the TCP domain to the R domain. The online TBLAST search confirmed its homology with *CYC/TB1*-like genes. As expected, the attempts to obtain the full-length cDNA of this sequence starting from the RNA extracted from an inflorescence of *O. italica* failed. In fact, the absence of an assembled transcript in the inflorescence transcriptome of *O. italica* corresponding to *OitaTB1* suggests that this gene is not expressed or it is expressed at very low levels and/or only in specific tissues of the inflorescence of *O. italica* at the stage of development examined. Further attempts to amplify the full-length cDNA of *OitaTB1* starting from RNA extracted from various tissues of the inflorescence or from leaf tissue did not give positive results. However, the *OitaTB1* sequence was used for all the subsequent analyses.

### Phylogeny, conserved motifs and evolutionary analysis

**Figure 1:**
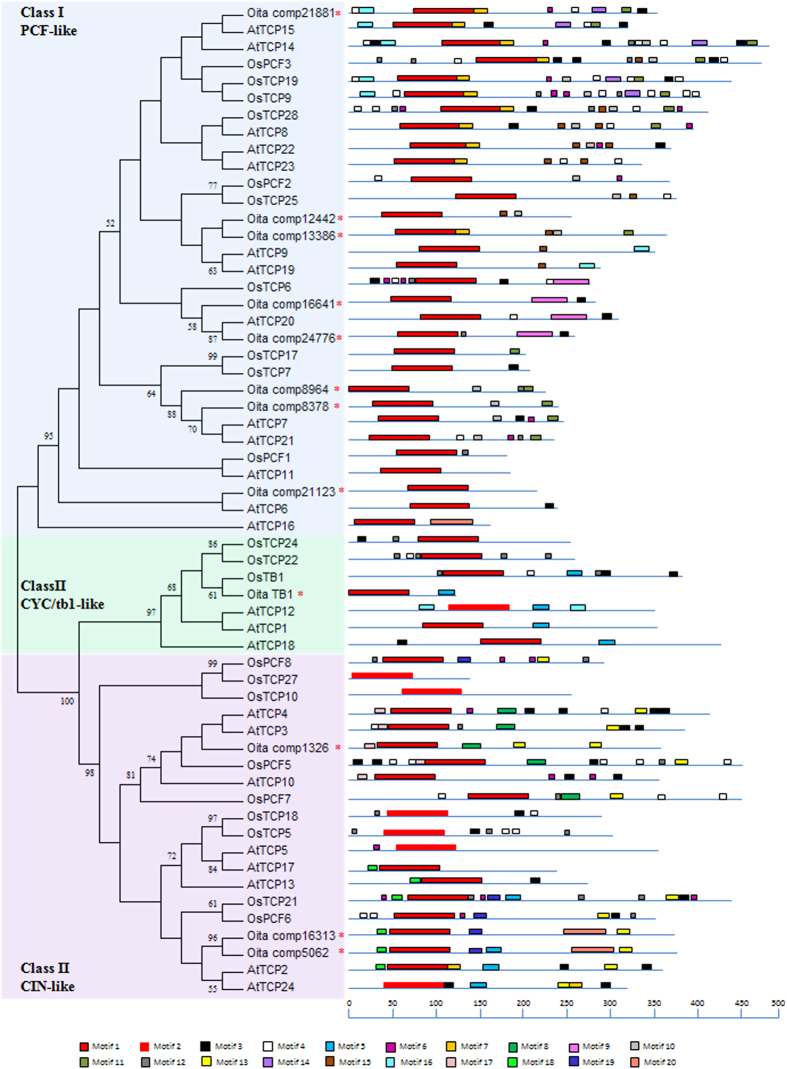
The NJ tree and graphical view of the conserved domains of the TCP proteins examined . The NJ tree was obtained from the amino acid alignment of the TCP domain of *Orchis italica*, *Arabidopsis thaliana* and *Oryza sativa* (Supplementary Table S1). The bootstrap percentages >50% are shown next to the branches. The red asterisks indicate the sequences of *O. italica*. The graph of the conserved domains and the corresponding legend were obtained from the MEME search using the full length of the TCP proteins. The scale below the conserved domains indicates the amino acid position.

The Neighbor-Joining (NJ) tree obtained from the multiple amino acid alignment of the TCP domain encoded by the selected transcripts of *O. italica* (12 sequences), *A. thaliana* (24 sequences), and *O. sativa* (22 sequences) is shown in Fig. 1. A high bootstrap value (95%) supports the class I PCF-like group that includes eight *TCP* transcripts of *O. italica*. Similarly, the class II TCP group is statistically well supported (bootstrap value 100%). It is divided into the CIN-like group (bootstrap value 98%) that includes three *TCP* transcripts of *O. italica* and the CYC/TB1-like group (bootstrap value 97%) that includes *OitaTB1*.

The generally dispersed positions of the different TCPs of *O. italica* among the branches of the tree confirm the hypothesis of the expansion of the TCP family before the divergence of the lineages examined, as observed in some dicot species^37^,^38^,^39^. The robustness of the NJ tree was confirmed by the topology of the Minimum Evolution and the Maximum Likelihood trees (Supplementary Figure S2 and S3, respectively), both showing branch patterns in agreement with the one obtained from the NJ analysis. In addition, the NJ tree inferred from the amino acid alignment of the TCP domains of Orchidaceae is in agreement with the classification of the TCP proteins (Supplementary Table S1 and Supplementary Figure S4). Similarly, the topology of the NJ tree obtained from the amino acid alignment of the TCP domains of *O. italica*, *P. equestris*, *P. hybrid cultivar*, *D. hybrid cultivar*, *A. thaliana* and *O. sativa* (Supplementary Figure S5) consistently distinguishes the different groups of the TCP proteins.

The amino acid sequences of the TCP proteins of *O. italica*, *A. thaliana,* and *O. sativa* were scanned to search for conserved shared domains using the motif-based sequence analysis tool MEME, and the results are shown in Fig. 1 and Supplementary Figure S6. The pattern distribution of the shared motifs is in general agreement with the phylogeny inferred from the alignment of the TCP domain. All the sequences share the TCP domain, included in Motif 1 and 2. The R domain (Motif 5) is shared by 9 sequences belonging to the class II TCP family, both CYC/TB1-like and CIN-like. Among them, two are sequences of *O. italica*, one CIN-like (comp5062) and one CYC/TB1-like (*OitaTB1*). Another interesting motif shared by 12 sequences of the CIN-like group (Motif 13) corresponds to the amino acid stretch encoded by the target site of the microRNA miR319^12^,^13^,^14^,^15^. This motif is present in three sequences of *O. italica* (comp5062, comp1326 and comp16313). Many other motifs are restricted to specific sub-groups of the tree, suggesting that they might contribute to the specific function of the different TCP proteins. For example, Motif 9 is present in OsTCP6, AtTCP20 and in two sequences of *O. italica*, comp16641 and comp24776; Motif 8 is shared by AtTCP3, AtTCP4, OsPCF5, OsPCF7 and comp1326. Scanning the identified consensus motifs against the database of protein domains PROSITE^52^ resulted in positive hits with the TCP domain or with other regions of the TCP proteins with unknown functions, or in no positive matches. In conclusion, excluding the TCP domain, the functions of the other conserved motifs identified are still unknown.

The estimates of the non-synonymous (Ka) and synonymous (Ks) substitution rates was performed on the putative orthologous *TCP* nucleotide sequences of *O. italica* and *P. equestris* to evaluate the evolutionary constraints acting on these sequences. The Ka/Ks ratio equal to 1 indicates neutral selection, while values lower or higher than 1 reveal purifying or positive selection, respectively^53^. The search for the best reciprocal BLASTP hits between the TCP sequences of *O. italica* and *P. equestris* gave significant scores for 10 pairs of sequences. The results of the evolutionary analysis are summarized in Table 1. Among the sequences examined, the mean Ka/Ks value is 0.222, and the pair-wise comparisons reveal that a strong purifying selection is acting on the nucleotide sequences encoding the TCP domain in *O. italica* and *P. equestris*. However, the comparison between the sequence comp16641 of *O. italica* and PEQU28429 of *P. equestris* resulted in a Ka/Ks value much higher than all the other comparisons and higher than 1, even though the p value was not significant. As shown in Fig. 1 and reported in Supplementary Table S1, the transcripts comp16641 of *O. italica* and PEQU28429 of *P. equestris* are related to the TCP factor AtTCP20 of *A. thaliana*. AtTCP20 belongs to the class I PCF-like group. It is expressed in different organs and developmental stages and is involved in cell division and elongation, which must be coordinated for normal plant development and differentiation^54^. The Ka/Ks value detected between the transcripts comp16641 of *O. italica* and PEQU28429 of *P. equestris* suggests a possible relaxation of the selective constraints acting on these sequences in orchids, which might drive the evolution of morphological innovations.

### MicroRNA target sites and expression analysis

To check for the presence of target sites of specific microRNAs (miRNAs) expressed in the inflorescence, the selected transcripts of *O. italica* encoding TCP-like proteins were scanned with the psRNATarget online tool^55^ using the inflorescence miRNAs of *O. italica*^47^ as queries. The results of this *in silico* analysis predicted for three different transcripts of *O. italica* belonging to the class II CIN-like group (comp5062, comp1326 and comp16313) the presence of a putative cleavage site for miR319, in agreement with the results obtained from the analysis of the shared conserved motifs. Phylogenetic analysis shows that comp5062 and comp16313 of *O. italica* are grouped with AtTCP2 and AtTCP24 of *A. thaliana*, comp1326 with AtTCP3 and AtTCP4 of *A. thaliana* and OsPCF5 of *Oryza sativa* (Fig. 1). All of these transcripts have been described as targets of miR319 and are involved in different developmental processes, from petal and leaf formation to senescence and response to different stresses^12^,^13^,^14^,^15^,^16^.

**Figure 2:**
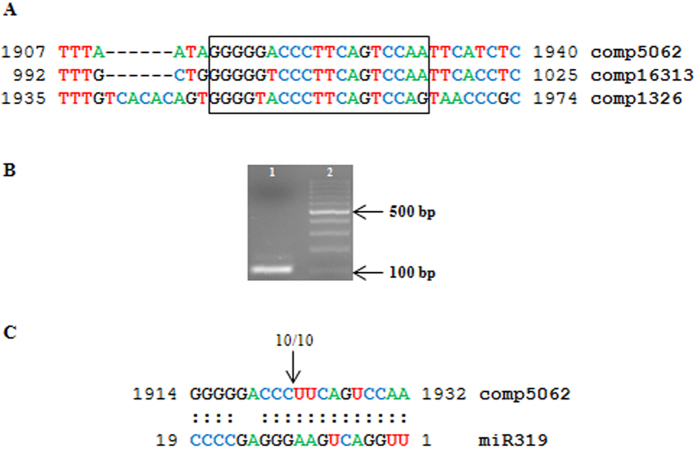
The cleavage site of miR319 on the TCP transcript of *O. italica* comp5062 . (**A**) The nucleotide alignment of the *in silico* predicted target site (highlighted in the box) of miR319 on three *TCP* transcripts of *O. italica*. Numbers refer to the nucleotide positions on the transcript. (**B**) Agarose gel electrophoresis of the modified 5′RACE experiment on the *TCP* transcript comp5062 (lane 1) and the 100 bp Ladder (Fermentas). (**C**) Alignment of the target site in the transcript comp5062 and the miR319. The arrow and the numbers above the sequences indicate the cleavage site and the number of sequenced clones that revealed the cleavage in that position, respectively.

The validation of the miRNA cleavage was performed by conducting a modified 5′-RACE experiment. Cleavage was confirmed for the transcript comp5062 (Fig. 2B,C), whereas for the transcripts comp1326 and comp16316, no cleavage fragment of the expected size was detected. The apparent absence of the cleaving activity of miR319 on comp1326 and 16313 might be due to the sequence divergence in the upstream and downstream regions surrounding the predicted cleavage site.

**Figure 3:**
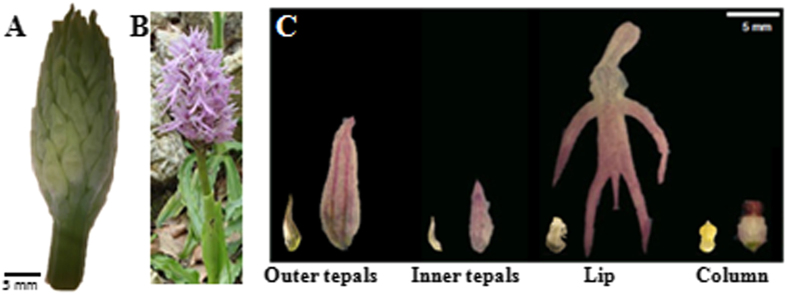
The Mediterranean orchid Orchis *italica* . (**A**) An inflorescence at the early stage; (**B**) An inflorescence at the late stage, after anthesis. (**C**) Tepals (outer and inner), lip and column collected from a single floret of the early (left) and late (right) inflorescence of *O. italica*.

**Figure 4:**
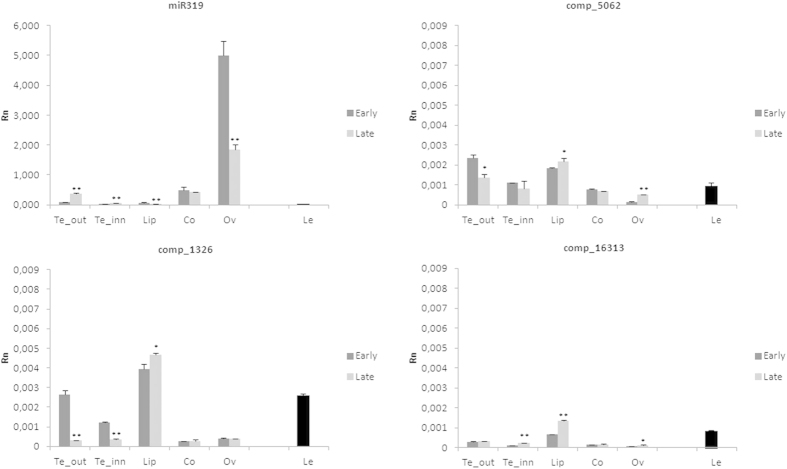
The relative expression of *TCP* transcripts comp5062, comp1326 and comp16313 and of miR319 in different floral tissues and in leaves of *O. italica*. The bars indicate standard deviation. The asterisks indicate statistically significant differences between the relative expression of the early and the late stages (*p < 0.05, **p < 0.01). Te_out, outer tepals; Te_inn, inner tepals; Lip, labellum; Co, column; Ov, ovary; Le, leaf. Rn, relative normalized expression.

The analysis of the expression patterns is useful to hypothesize possible gene functions and overlapping expression profiles indicate probable functional redundancy. The expression analysis of miR319 and of its putative target transcripts in different tissues of the inflorescence of *O. italica* at two developmental stages (Fig. 3C) was evaluated by real time PCR. The early stage corresponds to floral buds with a diameter of approximately 9 mm (Fig. 3A) and the late stage to completely opened flowers after anthesis (Fig. 3B). In both stages, cell division is completed, and the flower organs are completely formed; however, in the early stage, cells are still elongating. Figure 4 shows the relative expression of the transcripts comp5062, comp1326, comp16316 and miR319 in the outer and inner tepal, lip, column, unpollinated ovary and in leaf tissue. The expression patterns of these three transcripts in the tissues of *O. italica* at the two different stages examined are similar but with different expression levels. In the inflorescence, they are mainly expressed in the organs of the perianth (tepals and lip) with some difference between the early and late stage. In the column and ovary, the expression of the three transcripts is generally lower than in the other tissues, including leaves. In contrast, the expression pattern of miR319 shows the opposite trend, with the highest expression levels observed in the column and ovary. The observed values of the relative expression of miR319 are negatively correlated with those of its three putative targets; however, the only statistically significant Pearson correlation coefficient (*r* = –0.61, *p* < 0.05) is relative to the transcript comp5062. This result, together with the failure to detect specific fragments cleaved by miR319 for the transcripts comp1326 and comp16316, suggests the existence of different regulators of the expression of these two TCP transcripts in the tissues of *O. italica* at the stages examined. Our results demonstrate for the first time the activity of miR319 on a *TCP* target in the floral tissues of a monocot species. In the model dicot species *Arabidopsis*, miR319 has been demonstrated to have a critical role in the development of petal and stamens, through the cleavage of a CIN-like *TCP* target^15^. The differences of the expression pattern of the transcript comp5062 in the inflorescence tissues of *O. italica* and its regulation by miR319 make this *TCP* transcript a good candidate gene involved in the development of the floral phenotype of *O. italica* and suggest a possible conserved function of miR319 in flower development of dicots and monocots.

**Figure 5:**
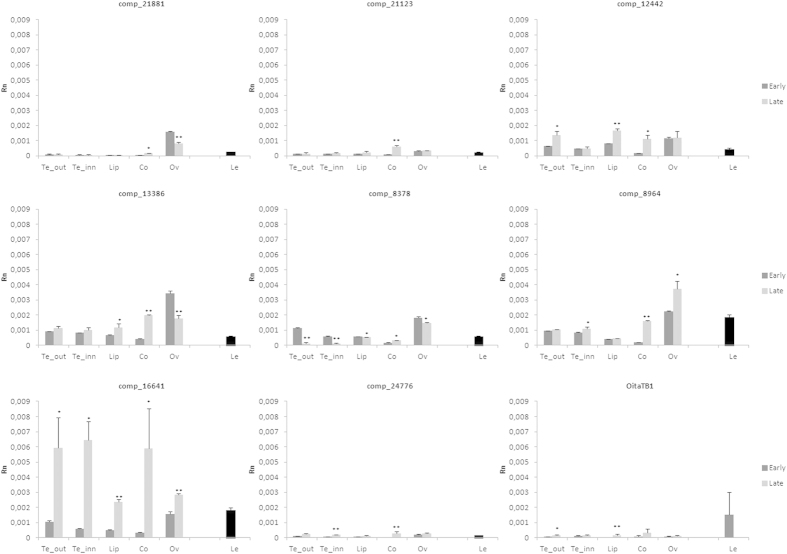
The relative expression of nine TCP transcripts in different floral tissues and in leaves of *O. italica*. The bars indicate standard deviation. The asterisks indicate statistically significant differences between the relative expression of the early and the late stages (*p < 0.05, **p < 0.01). Te_out, outer tepals; Te_inn, inner tepals; Lip, labellum; Co, column; Ov, ovary; Le, leaf. Rn, relative normalized expression.

The different tissues of *O. italica* were examined to evaluate the expression pattern of the other identified transcripts that encode TCP proteins. The results reported in Fig. 5 show distinct expression profiles for some transcripts, suggesting their different roles in the various tissues and stages examined, while other transcripts share similar patterns. In particular, the transcript comp21881 is expressed almost exclusively in the ovary tissue and the transcript comp21123 in the column at the late stage, suggesting that they might have a specific function in these tissues. The transcript comp21881 is related to the gene AtTCP15 of *Arabidopsis* (Fig. 1), involved in many developmental pathways including gynoecium development^56^,^57^. The expression profile observed in *O. italica* suggests its functional conservation, at least for its possible role in the development of the female reproductive structures. The transcripts comp12442 and 13386, belonging to the same branch of the NJ tree, display generally overlapping profiles, being expressed in all the tissues examined, including leaves, with some differences in the ovary tissue. This suggests pleiotropic and redundant functions of these two transcripts in the tissues examined of *O. italica*, in agreement with the diffuse functional redundancy of the class I TCPs. The transcripts comp8378 and 8964 belong to the same branch of the NJ tree, and they display similar patterns at the early stage. In the late stage, differences are detectable in tepals and ovary, where the levels of comp8378 significantly decrease, whereas the levels of comp8964 increase markedly in column and ovary. These two transcripts are related to the genes AtTCP21 and AtTCP7 (Fig. 1). In *Arabidopsis* these two genes show not fully overlapping expression profiles and functions. AtTCP7 is mainly involved in leaf development and AtTCP21 is a component of the circadian clock^58^,^59^. The expression pattern of comp8378 and 8964 indicates their possible functional diversification also in *O. italica*. Despite the fact that the transcripts comp16641 and 24776 are included in the same branch of the NJ tree, their expression profiles are very different. The first is highly expressed in all the floral tissues at the late stage and in leaves; the latter is very weakly expressed in all the tissues examined, suggesting that its functions are performed in different organs and/or developmental stages. The transcript comp16641 shows the strongest variation of the expression profile between the two stages examined and, as previously discussed, it shows relaxation of the selective constraints acting on its coding region. These findings let hypothesize a possible sub- or neo-functionalization of comp16641 and suggest to investigate in more detail the possible role of this gene in the development and maintenance of floral structures in orchids. Finally, *OitaTB1* is expressed at a very low level in the inflorescence tissues and at a slightly higher level in leaves. *OitaTB1* seems to be phylogenetically close to *OsTB1* of *Oryza sativa* (Fig. 1, Supplementary Figure S2 and S5), a negative regulator of lateral branching^60^. It would be of great interest to ascertain whether *OitaTB1* is also involved in this developmental process in *O. italica* and in orchids in general.

## Conclusions

This study has led to the identification of 12 transcripts encoding TCP proteins in the Mediterranean orchid *O. italica*. This number is lower than the number of the *TCP* genes of the model species *A. thaliana* (24) or *O. sativa* (22) because the approach used in the present study was focused on the TCPs expressed in the inflorescence tissues. Other *TCP* transcripts in *O. italica* are possibly expressed in tissues and/or developmental stages different from those examined. However, the number of transcripts identified in *O. italica* is not much different from the number annotated in the genome of the orchid *P. equestris* (17), suggesting that in orchids there could be fewer *TCP* genes than in *Arabidopsis* and *Oryza*. The analysis of the expression profiles of the *TCP* transcripts of *O. italica* shows that some of them are expressed in all the tissues and developmental stages examined, suggesting their potential involvement in multiple pathways, whereas others are restricted to specific tissues, indicating a possible more specialized role. In addition, the presence of a cleavage site for miR319 on one *TCP* transcript of *O. italica* supports the existence of an evolutionary conserved role of this miRNA in regulating the activity of specific TCP factors.

## Methods

### Isolation of the *TCP* genes expressed in the inflorescence of *O. italica*

The sequences of the TCP DNA-binding domain of *Arabidopsis* and *Oryza* (Pfam PF03634) were used as query to perform a standalone TBLASTN (e-value 1e-003) against the non-redundant sequences of the inflorescence transcriptome of *O. italica*^46^. In addition, the transcripts of the inflorescence transcriptome of *O. italica* annotated as *TCP* genes were selected. The transcripts of *O. italica* with significant hits from the TBLASTN search and those previously annotated as *TCP* genes were checked for the presence of an ORF that exceeded the TCP domain, excluding those shorter than 500 bp from further analyses.

The coding sequences (CDSs) and predicted proteins of the genome of the orchid *Phalaenopsis equestris*^42^ were downloaded from the FTP site ftp://ftp.genomics.org.cn/from_BGISZ/20130120/. The coding sequences annotated as *TCP* genes were selected (Supplementary Table S1) and used for the subsequent analyses.

### Isolation of the class II CYC/TB1-like sequences of *O. italica*

To identify the nucleotide sequences coding for CYC/TB1-like proteins, the genomic DNA of *O. italica* was extracted from leaf tissue^61^. Using the three nucleotide sequences of *P. equestris* annotated as CYC/TB1-like proteins, a degenerate primer pair was designed to amplify the region from the TCP domain to the R domain (Supplementary Table S2). PCR amplification was conducted on 100 ng of genomic DNA of *O. italica* in a final volume of 50 μl. The reaction mixture contained 200 μM dNTPs, 0.4 μM of each primer, buffer 1X and 2.5 U HotMaster Taq polymerase (5Prime). The thermal cycle was the following: 94 °C for 3 min, 35 cycles of 94 °C for 30 sec, 54 °C for 20 sec, 65 °C for 2 min, followed by a final extension of 10 min at 65 °C.

The amplification product was cloned into the pGEM-T Easy vector (Promega), and the positive clones were sequenced using the plasmid primers T7 and SP6 and analyzed on an ABI 310 Automated Sequencer (Applied Biosystems).

### Phylogenetic analysis and identification of conserved motifs

The amino acid sequences of the TCP proteins of *Arabidopsis thaliana* (24 accessions) and *Oryza sativa* (22 accessions) were downloaded from the Plant TFDB (Supplementary Table S1). Multiple sequence alignment of the TCP conserved domains of the downloaded sequences and the corresponding regions of the TCP proteins of *O. italica* obtained from the virtual translation of the selected transcripts was performed with MUSCLE^62^ and then manually adjusted. The Neighbor-Joining (NJ), Minimum Evolution (ME) and Maximum Likelihood (ML) trees were constructed using MEGA 6.06^63^ with 1000 bootstrap replicates. The amino acid sequences of the TCP proteins of the orchids *Phalaenopsis hybrid cultivar* (5 accessions) and *Dendrobium hybrid cultivar* (1 accession) were downloaded from GenBank (Supplementary Table S1). The TCP domains of the orchids *O. italica*, *P. equestris*, *P. hybrid cultivar* and *D. hybrid cultivar* were aligned, and the NJ, ME and ML trees were constructed as described above.

The whole amino acid sequences of the TCP proteins of *O. italica*, *A. thaliana* and *O. sativa* were scanned for the presence of shared conserved motifs using the online tool MEME^64^, setting the parameters to any number of repetitions, the optimum width from 4 to 70 and the maximum number of motifs to 20.

### Evolutionary analysis

To identify the best reciprocal hits, a custom-made perl script was used to perform BLASTP search on the TCP sequences of *O. italica* and *P. equestris*. Based on the results obtained, pair-wise nucleotide alignment between the resulting orthologous nucleotide sequence encoding the TCP domain of *O. italica* and *P. equestris* was constructed and used to estimate the synonymous (Ks) and non-synonymous sites (Ka) substitution rates using the pair-wise approximate YN method^65^ implemented in the KaKs Calculator^66^.

### Search for miRNA targets and cleavage validation

The psRNATarget online tool^55^ was used to search for the presence of microRNA targets within the *TCP* transcripts of *O. italica*, using as the query the microRNAs expressed in the inflorescence of *O. italica*^47^ and setting stringent search parameters (maximum expectation 0.0).

Based on the predicted position of the putative miRNA cleavage sites on the *TCP* transcripts of *O.italica*, specific reverse primers were designed, all of them annealing downstream from the predicted putative miRNA target, and used to verify the presence of the cleavage product by performing a modified 5′-RACE experiment^67^. In brief, total RNA was extracted from the inflorescence of *O. italica* before anthesis (~9 mm diameter) using the Trizol Reagent (Ambion). After DNase treatment, RNA was quantified using the spectrophotometer Nanodrop 2000 (Thermo Scientific). The 5′ adaptor of the RLM-RACE GeneRace kit (Invitrogen) was ligated to the 5′-terminus of the extracted RNA (500 ng) without any enzymatic treatment to remove the 5′ cap. The RNA was then reverse transcribed, and the cDNA was amplified using the GeneRace 5′ Primer and the TCP-specific reverse primers (Supplementary Table S2). A second PCR amplification was performed on 1 μl of the first reaction using the nested adaptor forward primer and the nested TCP-specific reverse primers (Supplementary Table S2). The amplification products were cloned into the pGEM-T Easy vector (Promega), and 10 clones were sequenced as described above.

The specimens of *O. italica* used in the present work are present in the orchids collection of the Botanical Garden of Naples (Italy).

### Expression analysis

Total RNA was extracted from different tissues of *O. italica* following the protocol described above. In particular, outer and inner tepal, lip, column and unpollinated ovary were dissected from the inflorescence of *O. italica* before anthesis (defined as the early stage, ~9 mm diameter) and after anthesis (late stage). Although in the early stage, cell division has been completed and flower organs formed, cell elongation is still occurring. In addition, total RNA was extracted from leaf tissue. RNA (350 ng) was reverse transcribed using the Advantage RT-PCR kit (Clontech) and an oligo dT primer.

Using the 5.8 S RNA as the endogenous control gene, real time PCR experiments were conducted on 30 ng of the first strand cDNA from each tissue using the primer pairs specific for the *TCP* transcripts of *O. italica* (Supplementary Table S2). The reactions were performed using the SYBR Green PCR Master Mix (Life Technologies) in technical triplicates and biological duplicates, and the thermal cycle was followed by a melting curve step^68^.

Stem-loop real time PCR experiments were conducted to evaluate the expression pattern of the microRNA miR319 of *O. italica* in all the tissues examined^69^. In brief, total RNA (150 ng) from each tissue was separately reverse transcribed using the miR319 and the 5.8 S stem-loop primers (Supplementary Table S2). The first strand cDNA (5 ng) was then amplified in technical triplicates and biological duplicates using the miR319 and 5.8 S specific primers and the stem-loop universal primer (Supplementary Table S2).

The PCR efficiency (E) and the optimal threshold cycle (C_T_) for each well were calculated using the Real Time PCR Miner online tool^70^. The mean relative expression ratio (Rn) and standard deviation of the *TCP* transcripts and of the miR319 in the different tissues was calculated using the 5.8 S RNA as the endogenous control gene by applying the formula Rn = (1 + E_target gene_)^−CT target gene^/(1 + E_control gene_)^−CT control gene^. Differences in relative expression levels of the TCP transcripts and of miR319 between and/or among the different tissues were assessed by the two-tailed t test and ANOVA followed by the Tukey HSD post hoc test. To exclude the presence of PCR artifacts, the real time PCR products of several samples were cloned and sequenced as described above.

## Additional Information

**How to cite this article**: Paolo, S. D. *et al.* Analysis of the *TCP* genes expressed in the inflorescence of the orchid *Orchis italica*. *Sci. Rep.* **5**, 16265; doi: 10.1038/srep16265 (2015).

## Supplementary Material

Supplementary Information

## Figures and Tables

**Table 1:** The evolutionary analysis of ten putative *TCP* orthologs between *O. italica* and *P. equestris*.

Sequence comparison	Ka	Ks	Ka/Ks	P-Value (Fisher)
comp24776_PEQU28429	0.03255	1.06064	0.030689	9.87E-18
comp21123_PEQU21260	0.149384	0.922071	0.16201	2.50E-05
comp8964_PEQU03822	0.07316	99	0.000739	0
comp12442_PEQU09751	0.011686	1.45184	0.008049	1.18E-22
comp13386_PEQU40981	0.022818	2.92432	0.007803	9.93E-30
comp1326_PEQU06996	0.114233	1.6597	0.068828	9.26E-28
comp5062_PEQU08153	0.073761	1.22877	0.060028	3.83E-19
comp16313_PEQU19547	0.111968	3.73888	0.029947	3.01E-17
comp16641_PEQU28429	4.05786	2.28581	1.77524	0.488247
OitaTB1_PEQU11715	0.249986	3.26082	0.076664	1.61E-26

The non-synonymous (Ka) and synonymous (Ks) substitution rates are calculated with the Yang and Nielsen (YN) method.
