# Supplementary material for: Analysis of the TCP genes expressed in the inflorescence of the orchid Orchis italica
Source: Sci Rep. 2015 Nov 4;5:16265. doi: 10.1038/srep16265 (PMC4632031; doi:10.1038/srep16265)
Supplement: Supplementary Information [file srep16265-s1.pdf]

Analysis of the *TCP* genes expressed in the inflorescence of the orchid *Orchis italica*

Sofia De Paolo, Luciano Gaudio, Serena Aceto*

Department of Biology, University of Naples Federico II, Napoli, Italy

*Corresponding author: Serena Aceto, via Mezzocannone 8, 80134 Napoli, Italy, serena.aceto@unina.it, +39 0812535190

Supplementary Figure S1

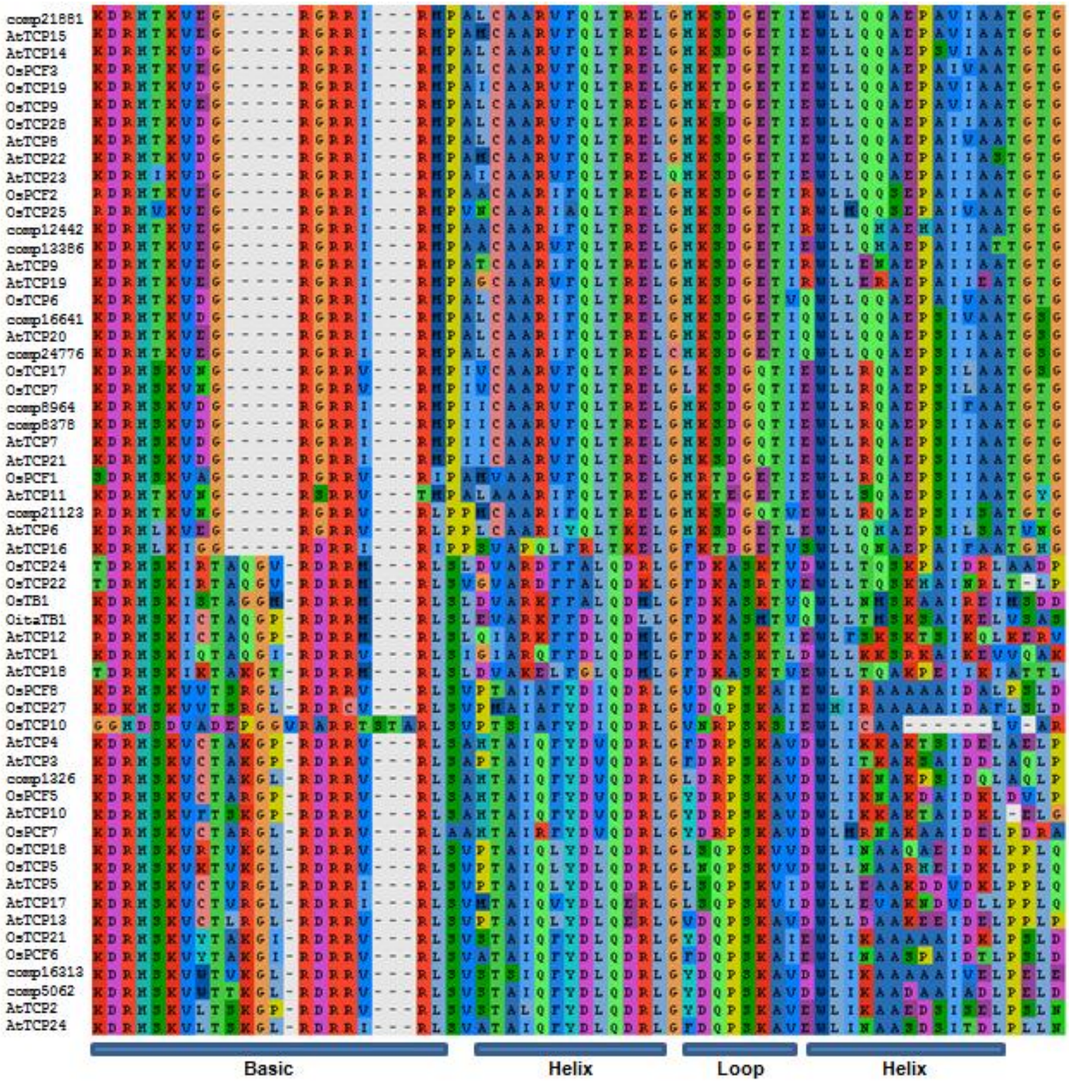

Figure S1 Multiple amino acid alignment of the TCP domains of *Orchis italica*, *Arabidopsis thaliana* and *Oryza sativa*.

Supplementary Figure S2

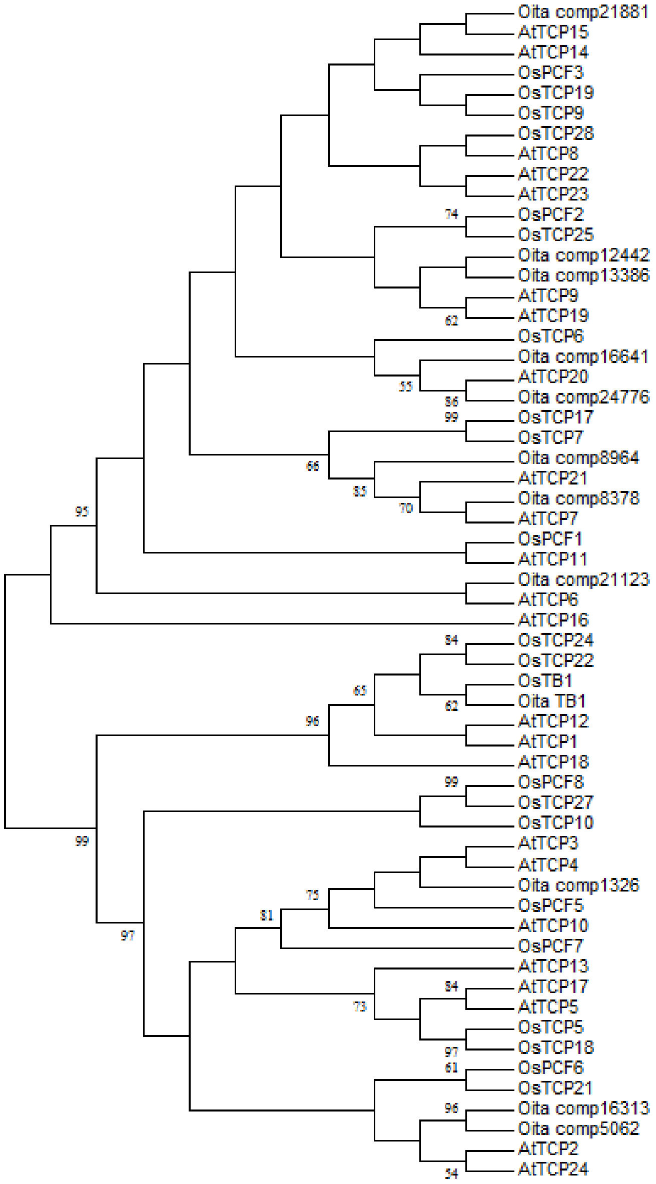

Figure S2 The Minimum Evolution tree obtained from the amino acid alignment of the TCP domain of *Orchis italica*, *Arabidopsis thaliana* and *Oryza sativa* (Supplementary Table S1). The bootstrap percentages > 50% are shown next to the branches.

Supplementary Figure S3

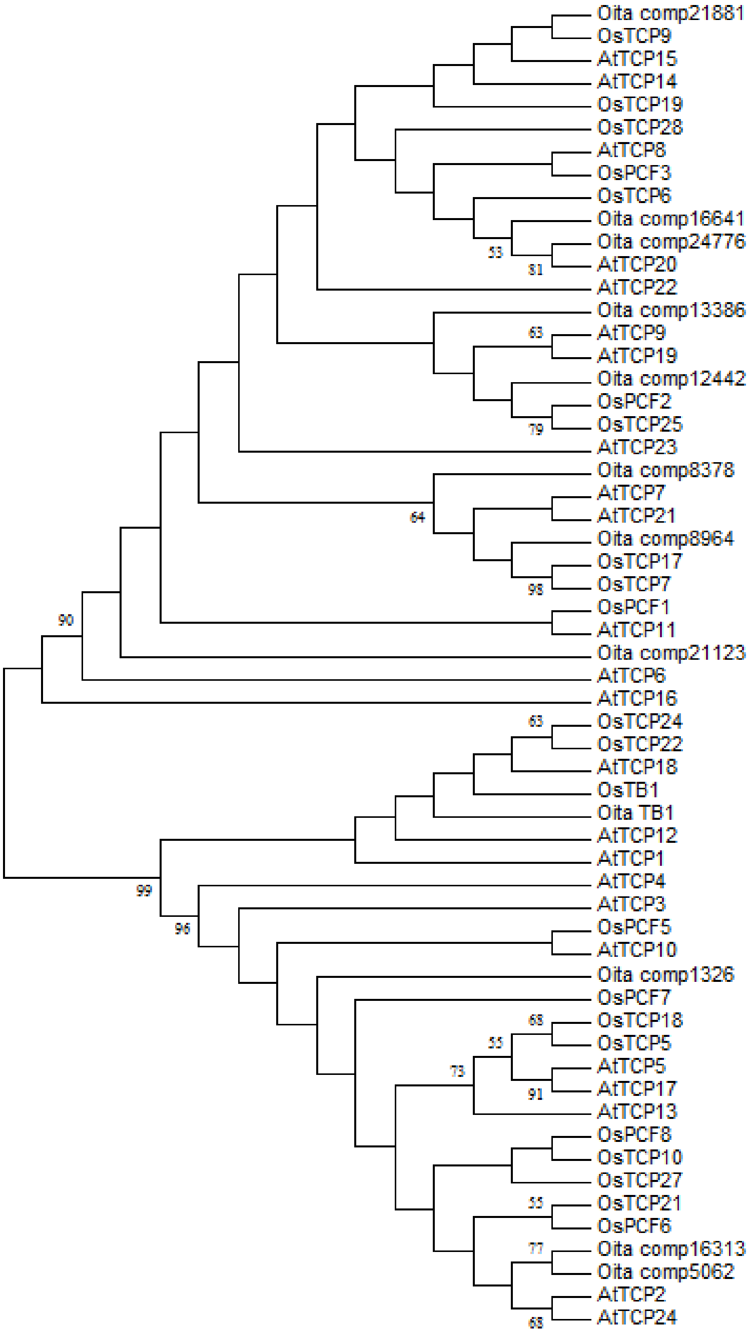

Figure S3 The Maximum Likelihood tree obtained from the amino acid alignment of the TCP domain of *Orchis italica*, *Arabidopsis thaliana* and *Oryza sativa* (Supplementary Table S1). The bootstrap percentages > 50% are shown next to the branches.

Supplementary Figure S4

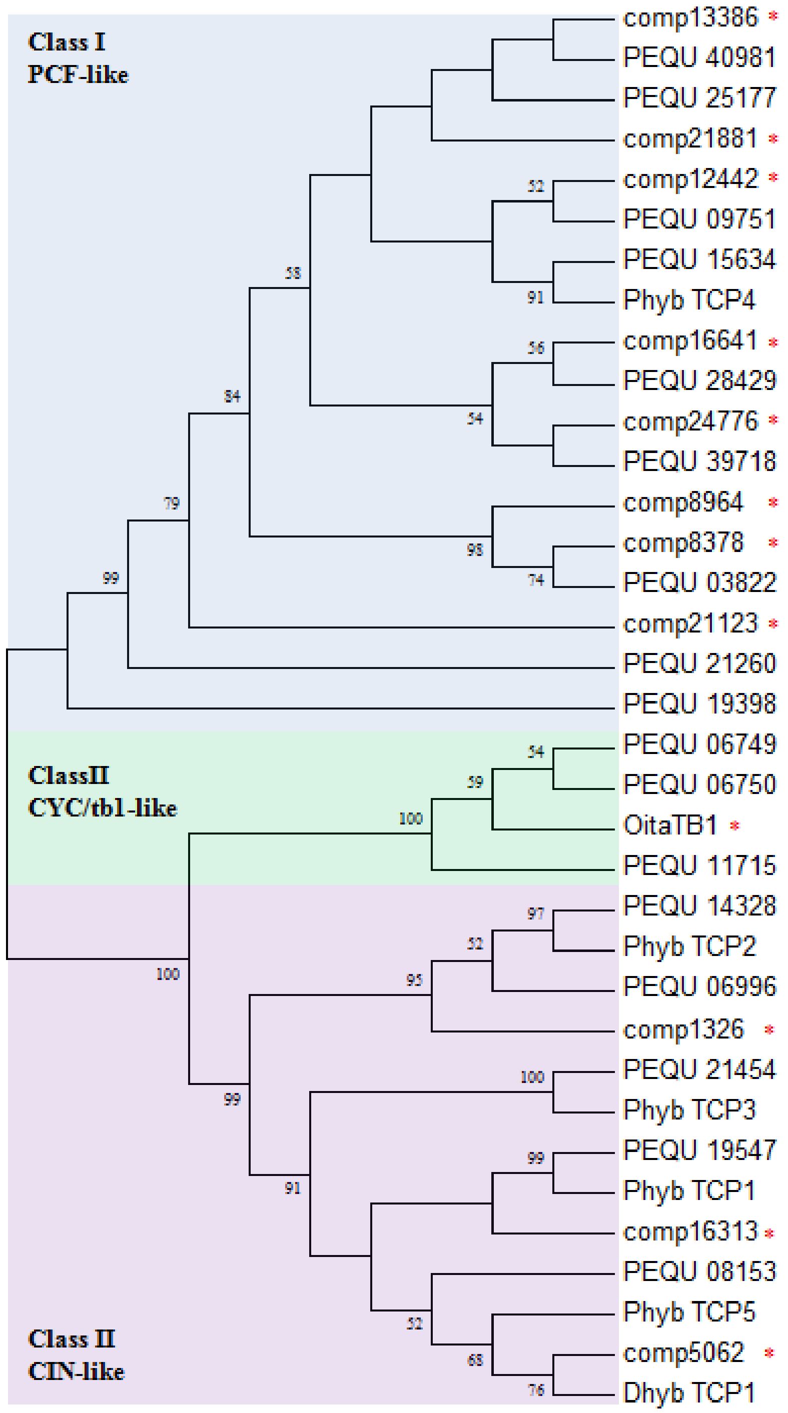

Figure S4 The NJ tree obtained from the amino acid alignment of the TCP domains of the orchids *Orchis italica*, *Dendrobium hybrid cultivar*, *Phalaenopsis equestris* and *P. hybrid cultivar*. Numbers indicate the bootstrap values, shown only when > 50%. The red asterisks indicate the sequences identified in the present study.

Supplementary Figure S5

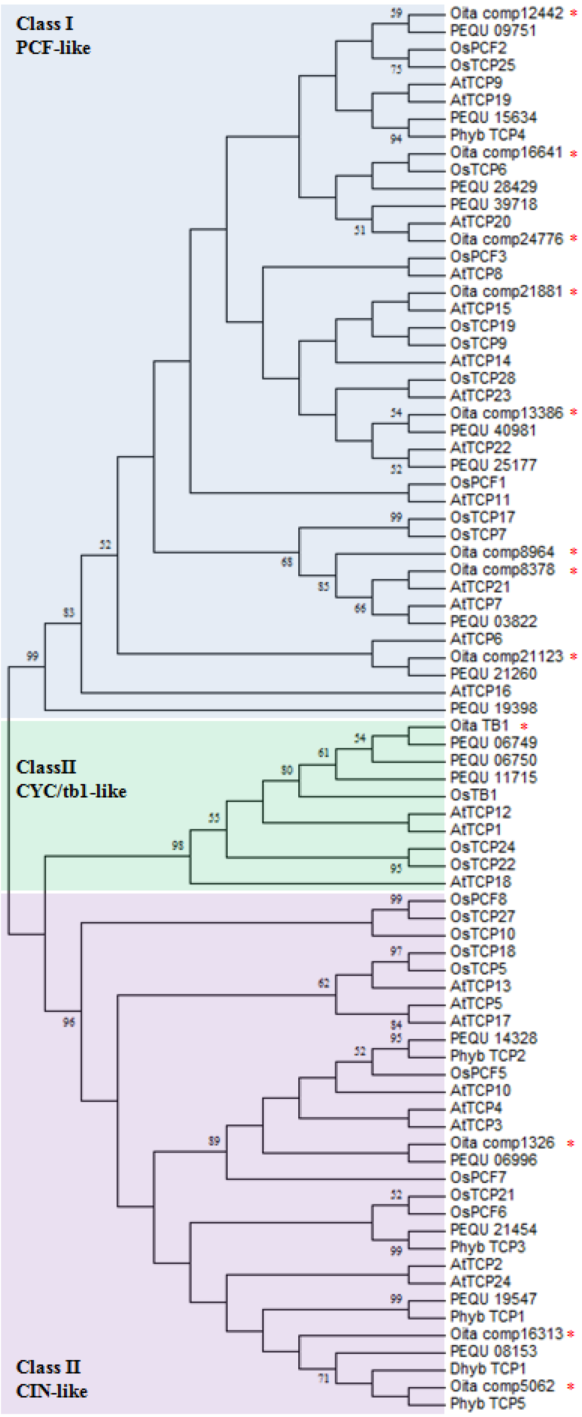

Figure S5 The NJ tree obtained from the amino acid alignment of the TCP domains of *Orchis italica*, *Dendrobium hybrid cultivar*, *Phalaenopsis equestris*, *P. hybrid cultivar*, *Arabidopsis thaliana* and *Oryza sativa*. Numbers indicate the bootstrap values, shown only when > 50%. The red asterisks indicate the sequences identified in the present study.

Supplementary Figure S6

Motif 1	[KR] [KR]D[RS] [HT] [KS] [DK] [RV]HT [KA] [VK] [GED]GR[GD]RR[IV]R[ML] [PS] [AV]L[CT]A[AI] [RQ] [VFI] [FY] [QD]L[TQ] [RD] [ER]LG[HF] [KD]S[DP] [GS] [EK] [TA] [IV] [ED]WL[LI]QQA[EK]P[AS]I[ID]A[AL]TG[TL]GTIPA[NS] [FA]S
Motif 2	RIVRVS[RV] [VT]FGGKDRHSKVCT[VS] [KR] GLRDRR[VI]RLSVPTAI[QA] [FL]YD[LI]QDRLG[LFV] [DNS]QPSK[VA] [IV] [ED]WL[IL]NAAK[AD] [AES]IDKLP
Motif 3	LHHH[HQ]HQHQx
Motif 4	GGGGGGGE
Motif 5	[KS]E[SL]R[AE]KARERA[RK]ERT[AKM] [EKA] [KE]
Motif 6	QQQQQQ
Motif 7	[ST]LN[IV] SLRSSGS[ST]LS[AS]
Motif 8	S[FL]LP[PAH]S[LM] [DG] [SA]D[SAR] [IV] [AS]D[TS] [IV]KS[FL]FP
Motif 9	[FM] [AF] [SA] [LIS] [LH] [GAS] [GA] [HAN] [AGHV] [AHNQ] [QA] [MI] PGLEL[GA]LSQ[DE]G[HNQ] [IV]GVL[NA] [PAS] [QHY] [SA] [LF] [SNT]Q[FI]Y[LHQ] [QH] [MV] [GV]
Motif 10	[PAG] [GA] [AGT] [FV]W[MA] [VL]P[AV]
Motif 11	[HN]L[GN] [LIM]LA[AS]L[NS] [AG] [YAG]
Motif 12	AAAAAA
Motif 13	[FS] [QN]RG[TP]LQS[NS] [SN] [PQS] [PS]L[FHI]
Motif 14	[VGM] [PSG] SG[LV]H[FL]MNF[PA]A[PT] [MPI] [AM] [ALF] [LF]
Motif 15	[PAQ]MWA[VFI]AP[GN]
Motif 16	[RKL] [PR] [NDT]F[PS]L[QE] [LI] [LY] [EDG] KKE[EKLS]QQ[CD]
Motif 17	IV[EA]VQGG[HG] [IH] [VI] [RV]
Motif 18	[SP]RI[IV]RVSRA[SF]G
Motif 19	[KD] [SD] [GTA] [CE] [SV]S[TA]SETSK[GS]S[VDT]
Motif 20	[HY] [LTY] [GF] [NS] [GNS] [PD] [NH] [MI] [GS] [MN] [GN] [MV] [MF] [APT] [YC] [NT] [PTV] [ATV] [NPS] [TS]G[EH] [HR]Q[DEM] [IMV] [FHQ] [QP] [FV]S[FIT]M[QT]DH[AV] [IP] [PS] [ATV] [SN] [APY] [PSV] [GPT] [IP] [DGS] [DGY] [ND] [LSY]N

Figure S6 The consensus sequence of the conserved motifs of the TCP proteins of *Orchis italica*, *Arabidopsis thaliana* and *Oryza sativa*.

Supplementary Data S1 The nucleotide sequence (in fasta format) of the *TCP* transcripts present in the inflorescence transcriptome of *Orchis italica*.

```
>comp8378_c0_seq1 len=1464 path=[13110815:0-1463]
CGCCTCTGGGAGTAATTAATCCCAACTTGCTCTCTCTCTATCTCTTTTTTCTCTTTCATC
TCCCAGTCTCCCGTGTTTCAGATTAGGGGTGATGACGACCGCGGCGGCCGACGGAGGGAC
GGATAATGGAAGCGAGAACGGGAAGACGAAGGCGTTGGTGGTGAAGAAGACGCCGGCGAA
GGACAGACACAGCAAGGTCGACGGACGGGGAAGGCGGATCCGGATGCCGATCATCTGCGC
GGCACGCGTATTCCAGCTAACCCGTGAGCTTGGCCACAAGTCCGACGGGCAGACTATCGA
GTGGCTTCTACGCCAGGCCGAGCCATCCATTATCGCGGCGACGGGCACCGGCACAACCCC
CGCATCCTTCTCCACCGCCTCCCTCTCCCTTCGTTCCGCCTCCTCCCCCATCGACCACAG
GCCCGGGACCCACCTTCTCCCGCAGCCGCTGGTGCCTTCTACCGCTCCCTTCTTCCTCGG
AAAGCGGCTCCGCACTGAGGAAGAGTCCGACGCCGATGCCAAAGACGAGGAGGTTGGCGC
CGGTGTCATGACTTATTCGCTGGGCTCCAGCGGCGGTTTCTGGGCCGTTCCGGCCCGGCC
GGACCTCGGACAGATGTGGAGCTTCACGGCCCCGGAGATCTTCATGCAGCCGCCAGCTAT
CTCAGGACGGTTTGCCGCCGCAGCGCACCTTGTCGGGGATTCATCGGCGGCCAGGATGGG
GACGAACTATGTTCCAATATCACAGGGCCACCTCAACCTGCTAGCCGCTATGTCAGGCCC
AGCTTCCATGGCCTCCACTAGGAGGGAAGACGATCCTCCCAGCTGATTCCCGAAACCGTC
TTGATCAAAGTTAGAGTTCGTTATTTCGGATAACAGAGGCATCGTCTTCCTCCTGTCCGA
TCTCTTATTCTCTCTCTAAGCCTGATTTGTTTCCACTGCTTTCATTCTTACTTTTTCATG
GCGTTAAATCTTCAAAGGGTCGGAGATGAGGAGCATCAATTGGGGAAAACAGCAAGCTCT
CGCCGCAGTGTCACCTCAGCTTGATGTTCAGAATGACCAGGCGTCATCAGCCTCTGATCT
TGATGATTTGGGGAAACTCCGTTGTGAATTCAGACCGCAGGTTTGTGCGCAATTCTTTTA
CGATTTCTTCATGATGAATTAATGTTTAATTTTAAGAGTGATTGTTTGGGGGGAAATCCA
TGGAGGTAGCTGTCGCTGTTCCAATTACGTATTTCTTCTCTCCATTTCTGGGTTTCATCT
TTGATTCTTCGAGCTGCCGGCGATGGGAATATTTGGGATTATGCATATAATTCAGGATTA
TTCCTGCTGAAGCACGGCTTTTGTACTTGCTGCAGTGTTCTAAAAGTAATCATTTTATTA
ACATTTATTTAACTGTATTATTAAGGCTTTATTTAATACTTTTAATAATTACTCTTAGTT
TTTAATTTGTTTTTGTGTCCGGGC
>comp8964_c0_seq1 len=1214 path=[14025538:0-1213]
GGCGAAGGACAGACACAGCAAGGTGGACGGCCGTGGAAGAAGGATCCGGATGCCGATAAT
ATGCGCCGCGCGTGTGTTCCAGCTCACGCGGGAGCTGGGCCATAAATCGGACGGTCAGAC
CATCGAGTGGCTCCTCCGACAGGCCGAGCCTTCCATCTTCGCCGCCACCGGAACAGGAAC
AACTCCGGCATCCTTCTCTACCGTCTCCACTTCCCTGCGCTCCTCCGCCTGCGCCCCGGT
GGAACACAAACCCGCCTCCTCCGTAGCCCCCTTCCACGGGGCCCACCTTCTGCCGCAATC
CATGGCGCCGGCGCCCTTCATACTCGGGAAGCGCATCCACTCCGAGGAAGACTCCGACGG
CGGCACTAAAGACAGCGGCTGTGGCGGGGCAGGCATAGCCTCCATGGCTCACACTTTAGG
TCCCGCCGGAGGTTTCTGGGCGGTTCCGGCGCGACAGGACTTCGGCCAGCTCTGGAGCTT
CGGGGCGCCGGAGATCATCATGCCGTCGCCGGCAGTCTCCGGGAGGTACGCCACCGCCGC
GCAGCTGATGGGGGAGGCGTCGGCGGCTAGGGTTGGGAACTACCTTCCCATCGCGCAGGG
CCACCTCAACCTCCTCGCCTCTTTGTCCGGTTCGCCTGCGGCGGCAGCCGCAGCGGCCGC
CGCCAGCAGGAGAAACGACGAGCACCGCTGATACTTAGGCCCCGGGATTTTTATCTTGAT
CGATTTCGCGCTGAAAATTCGAAGTTGTGAACTCGGAAGACGTAATTAGTTTTTCCTGCC
CTCTTTCTTCTCTATTAGATGTCTCTCACTCTTCCTCAAACCTCTCCTCGCTTCCTCCAT
GTTAGAACTGAAAATTCGGCAGCCGGAAGAAAATGTGGAGAAGATGAAGAAGAACATCAA
GATCATGAACTTTGGTTAATTTCTGCCGGGTAATTGCAGAGCTGGTCGTCGCTGGAATCG
ATTTCTGATGTTTGGAAGCTGTAGATTTGGAGTTCAGGATTTCTAGGGATAAAACACATG
GTAATTAAACTGTAGATATGAGTTCGATCGCCATTGTTGACAGCTGTGGTGGTGGTGGCG
GTGCCGGCGGCGGTGGTTGCAAGAGAGTTTTATCGATTTATTAAACTGTAGTGTATTTGC
TGTTGTTGGAGGCAATAATAGGCGATATGCATGTATTTCCGCATGTTTCGGGGTGAATTA
ATGGATGCAAGATC
>comp21881_c0_seq1 len=1796 path=[28475543:0-1795]
CACTCTTGCTCACTTCTGTCTCACTTTCGCTTTCAAAAACACACACACTTTCAGCATACC
TGGTATTCTCTCTCCCTCTCAAGCTATCTACTTGCCTTCAAATCCCCCCATTGCCATAAT
TAAATTTGAATTGGGTTTGAGTCGCCATGGAATGACATATTCTCTCTCGTCCTTCTCCTT
CCCTCTTCCCCATCTTCGCCTCTTCTGCATCTCTCTTCCCATCTTCTCTTCTGTGAAGAA
TTGAGTGTTCGTCCATCCCGCCCTCAATATGTCATCATGCTTCTCTATAAAATCACCACA
```

TATGCCCATATAAAACATATATACACGTGACTGTGTGTCTTCCTTTCTAGTTTTTAGATA
TTCATCTCCTTCCCTCATCGACCCCCGCCTCTCTGTCTCCCGGGCTGTGATCGATATGTT
CAAGTGTTTGATTTAGCTTTTAAAAAATTTAATATTTCTCCAGATTCTCATACAGTCGAA
GAAAATCAGTATCGAGATATTAAATTTTGTTATATATATAGTATTGAATCTCTTCATAAA
ATATCATGGCTTGAAGAAACTGCGGCATGGAGGGAGAAGACAGAGGAGGAGGCGGCGGTC
GCAGGCCCAACTTCCCTCTCCAGCTTCTCGAAAAGAAGGAATCTTTTGAAGATCCGAACT
GCTCGACATCGCTCGCCGCCTACCCCACCCTCGCCATCTCGTCCTCCGCCGCCGGGGCTG
GTCTTAGAGACGACCACCAACTCTCTGTTCCCGACGACAGCCACAAAAAGGTCCAGCCGA
AGCGCAGCTCCACGAAAGACCGTCATACCAAGGTCGAAGGCCGAGGGCGCCGCATCCGAA
TGCCGGCCCTCTGCGCCGCCCGCGTTTTCCAGCTCACCCGCGAGCTAGGTCACAAGTCCG
ACGGCGAGACCATTGAGTGGCTCCTCCAACAGGCCGAACCGGCGGTCATCGCAGCCACCG
GCACCGGCACTATCCCTGCCAATTTCACCTCCCTCAACATCTCCCTCCGCAGCTCAGGCT
CCACGATCTCCGCTGGCCCGTCCCACCACCTCCGTTCCGCCGCAGCAGCGCATTACTTCT
CCTCCGCCGCCCCCGCCTCCATGACGCCACTGCACCCTACCATCCGCCTTCGGTCCGACT
GGGACGTTTCTCCTTCCTCAGCCCCTTCTCTCCTCAACTTCCAAGACGTCAGCGAGGCGG
ACGCGTCCGGCAACATCCGAAAGCGGCGTTGGGATCAGGAGCTTAGCCAGCAGCAGCAGC
AGCAGGTGATGGCCAGCTACACAACGGCGACCTCACAAACGCAGATGCCGGCAACCATCT
GGACTGCAGGAGGTGGAGCGGGCGGCGAGGCGGTGTGGCCGTTTGCTCAGATGGGCGGAG
CGACCATGTTCCGAGGATCCATGTCCAGCGGTCTTCACTTTATGAATTTCCCTGCCACTC
CCATGGCTCTGCTTCCCGGGCAGCAGATCGGTCTTAGCTCCGGCGGCGGCGAGGGGCATT
TGGGGATTCTTGCAGCCCTTAACTCGTACAGGCCAGCCGGTTCGGCTCAGGAACCGTCGG
GTCATCATCAATATCATCATCAACAGCAGCATCACAGCAGCGGAGGGAGCGATAGGAATG
ATACGATGAGCACGAGTGACTCTTAGCTTCGCGATTTCCAAATGCAGTGTGCTACCTTCC
TCCCCAACTATTTTATAATTCCTTTCTATTCTCTTTTTATATCTTTCTAGCTTAATTTGT
TTTCCTTTTTTCAATTTTTATTTTCTACGTCCTGCGCGATTAGCTTAAAAAAAAAA
>comp21123_c0_seq1 len=920 path=[27873465:0-919]
CCCACATCTTCTTCAAGCCCTAGATTTTCGCCTACATTCCCGATCAGCGGCACCCGCCAC
TGACTCCTCCCCTACCACCGCCGCGGCAATCCTTCCTTGTTCTTTTTCCCTCGAGAAACC
CTCGCTATGCCCGGACCCACCTCGCTCTCTCTAATGCCCTCCCGATCGCCGTCTCCGTCG
CCGTCGCCGCAGCACTCGAACCTCACAATCCACAACCCAGCCATGCCCCTTCAGATACTT
CCGGAGGAAGATTATAGCTTACCAGCCCAGCGAGTCCGCGGGGGGAAGAAACGCTGCGGA
AGCGAGACTAGAGGCGGCGCCACCTTGTCGGGCTCGTCGAGGGACCGGCACACAAAGGTG
AACGGGCGTGGGAGGAGAGTTCGTCTTCCGCCGATGTGCGCGGCACGGATCTTCCAGCTT
ACGAGGGAACTCGGCCATAAATCCGACGGGCAGACCGTTGAGTGGCTCCTCCGCCAGGCG
GAACCCTCTATCATCTCCGCAACCGGAACCGGCATCGTCCCTCTCCCTGTCTCCAAGATC
TCAGCCAGCCCATCGCCATCATCTCCCCCGCCCCTTCCCACAAGCTTCTTCCAGTTCAGC
GGGATAATGGACTACAGCGCCGCCTTGGGAGGATTTGGATTCCCCGCCGCCGCCGCGGTT
GGCGAAGCCGTAGATGCCGGAATTGGATTCCGGCATGTGCCGTATTACACGGCCATGCTG
TTGCATCCGGCATCCGCCGAGGAGGAGGACAGCCGATCCGCCGTCGCGCAACCGCAGGAA
GATTAGCTGATTATTTTTTTATTTCGAAGCAGCGATGGAATTTTTGGGAGGTTGCAACGC
TGTCAATATCAGAAAAAAAGCAGCTTAAGGTATCGCGTTTTCATATCACGTTCTAGTTATA
TTTACTATTTACCCGCAGAT
>comp16641_c0_seq1 len=1654 path=[23704865:0-1653]
AATGGCGAAGACTCGGGAGAGAGGGAGGAGGTTAGAAATTATAGTACAGTAAAAGTCAAT
GTAAAATGGAGGAGAGAGAGAGCAGTAGCAGCAGTTTCTGCAGTACCTGCTGTAGCTGCA
GGCTGCAGCAGCATGCAGTTTACATCTTCGGTGCAAGATTCAAGGGATAAAACACAAGTC
TCTTTCGCCCAATCACTGTTGCTTTCTTCCACCCTCTTTTTTTTTTCTCTCTAAAATCCT
TGTCAAATCCCAATCCTCGCTGCCCAATTCGGCTCCTCCAGGCATCATCCATGGCTGCCG
CCCCTTCTCTTTCCCTCGCTCTCTGAGACGCGCAATCGTCCCGGAGAGGCAGAACCCGCC
GCCGCTCATCTATTTTCCGATGGATCCAAAGGGTTCGTCCGAGCAGGACAAGGGCGAGAC
ACGGAGAGAGCTGCAGATTGTGATTTCGAACTCGGAGAGGGAATTCCCTCCCGTGGCCTC
GTCCACGCCGGAGAAAGATGATCAGAAGAAGCAGCTTGCTCCCAAGAGAAGCTCCAACAA
GGATCGGCACACCAAAGTCGACGGACGCGGCCGGAGAATCCGTATGCCGGCGCTCTGCGC
CGCCCGAATCTTTCAGCTGACGCGGGAGCTCGGTCACAAATCCGATGGTGAGACCATCCA
GTGGCTCCTTCAGCAGGCGGAGCCCTCCATCGTCGCTGCCACCGGGTCAGGCACCATCCC
TGCCTCAGCCCTCACCTCCGCCCCCTCTTCCAACTCCCACCCCTCCACCTCCGCCGGCCT
CCACCCCAAGCTCAGCGATCATCCTACCTCCGGATCTGCCGCAGCTTCCAGACCCAATTG
GGCGGCCGGCCTCTGGCCTCCCCCGGTCCCCAGTTTCGGCAGCCACGGATTCCTCCCCAT
GACGAATCTCGCAGGGAACAGCGAAGGCCCTTCCGGAGGAGGGTTTATGCAGAGAATTGG
CCTGCCGGGGTTGGAGCTTCCCACGGCCAACCTAGGGCAGATGAGCTTCGCCTCACTGTT

GGGCGGCCATGGTCAGCAGATCCCCGGCCTCGAACTGGCTCTCTCCCAGGACGGGCAAAT
CGGGGTTCTAAACCCTTACGCGCTGAATCAGTTCTATCTGCATATGGGACAGGGAAGAGG
GGCAGCAGGTGATGAACAATTACACCATCAACACCAGCAGCAGCAGCAGCAACATAGCCA
AGGATCTCTGAACAAAGATGATTCTGATGGGTCGACTCAGTAGATGGAAAACTCCATTGA
TGGATTATAGTGGGTGCTGTGATCTTCATATGTCTAGATAATGGAGGAGACTATGAGAAT
AATCTGTTGGTAATGGTGTTTCATTGAATCAGTCCATCCTCCTCATCAAGGTAATCTCCC
TTGTCATGGTTGATTTTTATATCTGGTAGATGATTGAATGGAAAGGATGTGTTCACTATT
ACAATATTCTTCTTCTTCTCCATTCATATTACCATATACATGTGAGCTTCCTAGAATGAT
TTTGCTGCTTATCGTTTGTTTCAGAGATTGTTTATTTGCACATTTCATCAAAAGCCAGTT
GTTAGTTCTTTGTTATTTGATATGAAGACGATTGATTCACAAGTGCAAATGTACATTTTT
TGTAGTAGATTGGGCTAGATCGGAAGAGCGTCGT
>comp13386_c0_seq1 len=1881 path=[20030337:0-1880]
GATAAACGTAAATGGCTGCTCTCCTGGGGATCGGCATCGCCATTAGGGCTTTGAAAGCTT
GTCCGGCGGCATCAAGTCAATCACAAAAATGGAAACAAGGATGGTGATTGAGACGAAGCT
GTGGAGGAAAAGGCAAGTAGAGGGGTGAGTTTCTCTCTCTTTTCCTCTCTCTCCCTCTCT
CTCTCGCTCGGCTTCCTGCTCAGCTGCCATGGCGATCTCATAGATCTATTTATCATATAT
ATATATATTACCCTCATTACTTACGAGGCTCCACGAGTGGCCTACAGCCCCATACTGTTG
CCGTCTTTCTCCAGCTATTCGGAAGACATGGCTCTGCCCGCCATGGCGCAACCATGAGAG
AGAAAAAGCTGCGAGATTATCGGGAATAGGTCTGAATTAAGAAATCCGGTAGACCTCCTC
CTTCTCCGGCGGAATCGAGTTCTCAGAGTCAAAGCTTTTCGTATTACTTGCCCAGCTCAC
TCTGCGGAAGCGGAGAAAAGAGTCAACAGGAATAAGGCCGGAGCACGTGTTTCACGAAAC
CCTAACCCCTTCTTCTCCACTCTGTTTTATGTTTATTTTAATTTCGTCAGCGAATTTATT
CCGAGGATTTCATGTGGCTGGGAAGAAATAACGAGGACGAAATTTTGGGAAGAAATAGGA
ATAAGATTGAGGGGTTTCAGGATTTTTTTCCATTCCGATTTGTAAAGAATGGAGCATGCC
GGTGGAAATAATGGAATCCACGCGCCGTCGCCGGATCCGGCGGCGACGGCAGCGCGATTC
GCTGGATCCGGGCACGTTGACTCATCACTTGCAATTTCAACAGCGAATCCGACACCGGCT
CAGGATCCTAAGAAGACGGCGTTGGGGACGGCGAAGCGGTCGTCGAAGGACCGGCACACG
AAGGTTGAGGGCCGTGGGAGGCGCATCCGCATGCCGGCTGCGTGTGCGGCGCGGGTATTC
CAGCTCACCCGCGAGCTTGGGCATAAATCAGACGGGGAGACGATCGAGTGGCTGCTGCAG
CATGCGGAGCCGGCCATCATCGCGACCACCGGAACCGGCACTATTCCCGCCAATTTCTCC
TCTCTGAACGTCTCCCTCCGCAGCAGCAGTTCTTCCCACTCCGCGCCGCCGTCCAAATCT
GCCCCGCATTCCTTCCACGCTCTCGCCCACCACTACGAACTCGTCGACCCCTCCTCCATG
CTCGCCTTCCAGCCCGACGGCGCCCTGGGCCACGATTCGGCGCCCGACACTTACATGCGA
AAACGGTTCAGAGGAGATCCTTTCAAAGAGGAGCAAGACGACGCCGGCTCCGGTGGCGCC
AGCGGCGCCCAGTCGGCTTCTCCGCCGAAATCCGGGTCCCGTCCCGTCCTCCCCGCTGCC
GCGATGTGGGCCGTGGCTCCTGGGAGCAGCGCAGGGCCTGGGGCGTTCTGGATGTTACCC
GTGTCGGCGGGATCTTCCACCCCTGCCCCGGAGACTTCTTCACTGTGGACGTTCCCGGCG
GCGCCAGGCGCGAACACTTTGCAGCCGGCGCCCTTGCAGTTTATGTCGAGGATCAACATC
CCAGGGGTGGAGTTTCAGAGCGGCCGAGGCGTCAGCGGGATGCCGATGGGGTCGATGTTT
CCGGCGGCCCAGCATCTGGGGCTAGGGATTGCTGATTCGAACCTTGGAATGCTGGGTGCT
ATTAATGCTTATAATCGTAGTGGCGGCGGCGGCAGCGGTGAGATGATGAATTCCGATCAT
GGTCCGGCGATGGATAGGCGCCGGCAGCAGCAGCAAGGTTCGGAAAGTGGAGGCGGAGAT
CGGCCTAGCAACTCTCAGTAGAGGGAGATCAAATGGTAAGTGGGAAGATTAATTCCCGCT
GCAACTGCATACTAGCACTGC
>comp24776_c0_seq1 len=1251 path=[30640444:0-1250]
CAGGGGGTGTGCATCTTCATCCGCTCTCTCCAAGCCTTCTTCAACTCCCAGAGTCCCACC
CATCCCTCTTATTTCTCCCACGATGGATTCCAAGGCTTCTCCCAAGCTCCCGCACGAGAG
CCAAGATGAGGTGGAGCCCCAAAGAGACATTCGAGAGCTCCGGATGGTGATTTCCGCCCC
AGCTTCAGGAGCAGACATAGATTCGACTTTTGTAGTTGAGAAAGATGATCAACGGAAGCA
TCTTGCACCCAATAGAAGCTCAAACAAAGACCGGCACACCAAGGTTGAAGGCCGTGGCCG
GAGGATACGGATGCCGGCTCTCTGCGCCGCCCGGATCTTCCAGCTTACCCGCGAGCTATG
CCACAAATCTGACGGTGAGACTATCCAGTGGCTTCTGCAACAGGCTGAACCCTCAATTAT
CGCTGCCACGGGATCCGGCACCATCCCAGCCTCGGCCCTAGCAGCCGCTGCTGTCGCTAT
CCCCTCTTCTATCCCCCACCCCTCAATTCCCGCCGGCCTCCACCCGAGGCTTAACAACCT
GCCAGCAGCCTCGTCGGCTCGGCCCAATTGGGCTGCTGGTCTCTGGCAGCCGCCCGTCAC
TAGCTTTGGGCTCACAGGATTGGAGCTTCCTGGGGCAAATTTGGGTCAGATGAGCTTTGC
ATCATTGCTAAGTGGGCATGTACATCAGATGCCTGGGCTCGAGCTAGGACTTTCACAAGA
TGGACACATTGGGGTTTTGAACTCTCATTCTTTGAGCCAGTTCTATTTGCAGATGGGTCA
GGGAAGAGCGGGAGGAAGTGATGAGCACTTGCAGCATCACCATCAACAGCACCAAAGCCA
GCGGGACAAGGATGATTCCGATTGCTCGAGTTAGCAAATATTTATTTGTTGTACTGAATT

TCTACCATGATATTTGGGGTGGAAGATGGAGGATGGCATTAAAGGTATCATATTGATCAG
AGGTACTTCTCCTTCCTCTGGCCAATAGGCTTGCATTCTCCGGATCGAGGAAGACGAGGG
GTTCTCATAAGGCTACGAAGAAGGCAAAAAAGTTCAAGAAACAAAAGCAAAAGTTGGCAG
AGGAAATAGCTAAGGTATGAGTTAAAATATTTATTGGAATAGAGGAAACGGTAAAGAGAC
AAAGAAAAAATATGAGAATTGGCAGTGTTTAGTCTGTAATCAAATAGATTGGATAGTTTTG
AGTCTGTAGTGTGTTGTAGTTTGTCGTAGTGTGTAGTTAGTTAGTCGCAGT
>comp12442_c0_seq1 len=850 path=[18888421:0-849]
CGACGCTCTTCCGATCTATTCCCTTTTTACTCCGTAAGCAGGGAGGAAGGAGCGAAAAAG
GTTTTCAACACCATGGAAGACGACATCAGAAGCACGACCGCCGAAGGCGGAGAGACGGCA
GCTCGCCTCCGGCAAATAGTCCCGAAACTGGAGCCGACGGATATAATCGGAGCGACGCTT
CCGATCATCAGGATGCCCTCAAGGCGCACCAAGGACCGGCATACGAAGGTAGAAGGAAGA
GGACGACGGATTCGGATGCCGGCAGCCTGCGCGGCGAGGATCTTCCAGCTCACCCGCGAG
CTCGGTCATAAATCTGACGGCGAGACCATCCGGTGGCTGCTTCAGCACGCGGAGCACGCC
ATCATAGCCGCGACTGGGACCGGAACTATACCTGCCATCGCCACCAACGTCAACGGAACT
CTCAAGATCCCCACGGAATCCCAGTCGGCCGGGACATCGGGGGTGGATGACGCTGCCGGA
AAGAGGAGGAGGAAGTTGCAGCCTACGCGAGGTTCCGATCATCCACTTCTCCAGGTTAGC
GGTTCGGTTTCGGTTCCAGCGGGTCTGGCTCCGATAGCGCCGATGTGGGCCATCGCCGGA
GGCACTGCAGGGATTACCGGTGCGTTAGTGCCTCCGGGGGCGGTATGGGTGGTGCCCCCT
GGTCATATGTGGGGGTTTCGGGCCGTGAGCATGTTGAACGGCGGCAGGCGCGGGGGTTCT
CCGGAGGTTGATACCGGGAAGCAGGAGCTGCAGTTAATGAGGGAGACAGCGGAGGTAGCA
CGGCCGGAGGAGGGAGACGAAGAAGATCAGCAACCGCCGGAAGCAGTGGTGGGGACAGAT
CGGAAGAGCA
>comp16313_c0_seq1 len=1507 path=[23350909:0-1506]
TCTTTTTCTTCCTCGGAGAAGAGGATAATTAAGAGATTGGTGCGGGGAGAGGGGGCATGA
GAGCAGAATGGAGGTGGAGGGGATCCACTCGAGCAAGCGGTCCAGGAGGGATGAGAACGT
GCCCCAAGCAGACGTGAAAGGCGTCGATGAACTCGTCTCATGGCAGAATGCATCTTCAAG
AATTATTAGGGTTTCGCGAATTTCCGGAGGTAAGGACCGCCACAGCAAGGTGTGGACGGT
GAAGGGGCTCCGAGACCGCCGCGTGCGTCTCTCTGTCTCCACTTCCATCCAGTTCTACGA
CCTACAAGACCGCCTCGGGTACGACCAGCCCAGCAAGGCCGTGGACTGGCTCATCAAGGC
GGCCGCCGCCGCCATCGTCGAACTCCCGGAGCTCGAAGGCCCATTTCCCGTCCCGCCGTT
CTTGGCACTTCCTGGTCGCGCCATTCTCTCCCAGAGATCTCTGGCCGGCGAGCAGCAGCT
GTCTCAGTCCAAATCGACCTGCAGCAGTGCCTCGGAGACCAGTAAGAGCTCGGTGCTGTC
TCTCTCCAGGTTCGAGAGTCTCTTCAACGAGAAGAATAAGGACCGTCAAAACCAGAACAA
GGAGAGCTCCTTCAGTGACCAGCTCACTGGCAGCGGCAACATCAGTGGTGGTACTCCGGC
AGCTGCAGTGGTGGATTTTGGCAGCAGTTCTTATCCGAGGCAGCTGAGGACGCTACCCCC
AAGTTCTACATCAGAGTACTTCAGGGAAGCTGGAAATTTTGGACATGGGAATCGAATTCC
CCAGCTTGAAACTAGTTTCTCTTCATTTTTGCATTATGGAAGCAGCCCTAATATGGGGAT
GGGGATGATGACATACAATCCAACTCCTTCAGGAGAGCATCAGGACATACATCAGTTTTC
CATCATGCAAGACCATGCAATTCCTGCATCACCTGTGCCACCTGGGGGAAATTCTAACCT
TAATGTCTCCGTTTCTTCCGGCATTGCCAGATTTGCTGGGGGGTCCCTTCAGTCCAATTC
ACCTCATCTACACCATCTTATGCATGGTGCAAACTTGCCCTTCTTCCTTAGCAGCACTAT
TCCAGTTGCAGCAGCAGCGGGAAGCTCGCTGGAGAACCAGTTCCCTGGTGGTTTCAATGG
CCATCTGCAGCTGTTCTGTAATGAGGCATACATGCAGCCGGAATTGAAGGAGGAAAGGAA
GGGTTGAAGCCTGTAATCTGCCGGATCCTACAGTTTTTCGTGGTCTGCCCTGGCGTGATG
TAGACGCCGAAGACAATCGCCCGGCGGATTATCTTCAGTTTCTGATGAGGCCTTCAAATG
CTATCAGTTTATCCTCTTTTAACTTGTTTCCATGAACTATGTGTGAGCCCAGTGCTCAGG
ATTCTTGCCCTGTTATTTGGTTTATGTAAAATCCCTAAGCTATCTATTTTGGTTTTCATA
TGCTGAGGAGAAAAAAGTTACTTTTCTTCTGCCTAGTGATATGGAAAAAAATTTGAACCT
TCAGCTC
>comp5062_c0_seq1 len=2394 path=[7899858:0-2393]
AAATCCCCGATCTGTACGTCTGTAGGTTAGCACTCCCATCACCCTAGGATATAGGGTAGT
AGGGCTATTCTTGTCGCGACTAGTAGATGACAAATTCGATAATTTGATGAATTCCAAGGG
TACTTCTGCGGTAACTTTTTGAACTATAAAGCCCAAGGCGGCGCACGGCGGCAAACGGCG
CAATCCCCGGTGAGATCGTCGTTTCTCTGCCGGGAACAGCGCTTCGATTGATCGAATCCC
GAGCTCTACTCTCCGGCTTCTACTTTATCGGGTTCTGCCCCTTCTTTTTGTTAGCCGGAG
AAATCCAGTTCTGGTCATGGTAGGTACAACGGAGGTCGGAGATAAAGCAGGAGAGTTGAT
CGAAGTTAAAGTCAATTCTTCCTATATAAGTTTGGATTAGAAACCAGAAGAAATGCTCCC
ATTTCCTGTCGTCTCCGAAACCCTAAATCTCAGATCCGAGCAACGAAGGCTCTCTCTCAC
TTTTTCTGAGCTTCTTTTCGTCTCGGCCTTATCCTGAAATAAATCCTTGCCGCTTTTTTC
TCTCGAAATTGTCGCCATCTTTGTACATAGAGCAGTCGTAGTGGTGAGGGCAGGCGATCG

CAGTTCGCTGGCGAGCGATCAGCGAGAGAGATGCTGCGTCGCAGGCGCCGGCCCAGCAAC
CCAGGCGGCCTGAGGATGACGCCGCTGGTAAAAGCGGCTCGTCGCTGACACATCTCGGCC
ACCACCCTATTTCCAGCGTCAAACGAGCTATTCTTTTTCTCACCTTCCCTCGAAAGCCTC
ATCAATGCAAAAGGACACGGTGAAAGTTGAATCCCTAAACGCTACCACTCACGCCCCATT
TCTCCATATAAGGAAGCAGGAGAAAGTTAAAAGCCAAAAAGGCCAAGTTCTTATTTAGTA
GGGGATAAGAGCGGGATATCGAGTGTAAAACCTTGGTTATCGGGGGAGCAGGGAGGGGCA
TGAGTGAGGGATGGAGGCGGAAGGGATCCACTCGATCAAGCGGCTTAGGAGAGACAAAGA
CGAGGAAAACGGGGACGTGAAGAGCGTCGCCGGTCTCCGACCGTGGCAGAACGCCTCGTC
GAGGATTATCAGGGTTTCCCGCGTTTCCGGCGGGAAGGACCGCCACAGTAAGGTGTGGAC
GACGAAGGGACTCCGAGACCGCCGCGTACGTCTCTCTGTTTCCACCGCCATCCAGTTCTA
CGACCTCCAGGACCGCCTTGGCTACGACCAGCCCAGTAAAGCGGTAGACTGGCTCATCAA
GGCTGCCGACGCTGCCATCGCCGATCTGCCCGAGCTTGATGGCGCATTTCCCATGCCACC
CACTATGGTTCTTCCCATTCGAGCAAATCTGCATGAGCGAGCAGCCGCCGGCGAACAGAA
AATCTCCCCGTCCAAATCCACGTGCAGCAGCGCGTCGGAGACAAGTAAGGGATCTACACT
ATCCCTTTCCAGGTACCAGGCGAGGGAGCGTGCTCGGCATAGGACGGCAAAAGAGAAGGA
GAACGATCAAGACGCATCCCATCATCAAAATCTCATCACGCACAGCTCCTTCACTGAACT
TCTCAATGCAGGCCCGACCGTTGAAGGGGCAGATTGCGGCACTGATTTTGGTCAAAAACA
TCTCAGGCCTCAGCTGTCATCTCCAACCGCCGATTACGTTGGTCAGTCTGGGATGTTTGG
GCAGACACATAGAATTCCACATTTGTTACCGGGTTATTCTACTCCCCTGATGCATCTAGG
GAACAATCCTCACATGGGAATGGGGATGATGGCATACAATACTGCTTCGACCGGAGAACA
TCAGGAAATGCAGCAGTTTTCTTTCATGCAGGATCATGTAATTCCCGTGTCAGCTCCAGG
GATTGACTATGATCTAAACGTCTCCATTTCTTCTGTTTTTAGTGGATTTAATAGGGGGAC
CCTTCAGTCCAATTCATCTCATCACCACCACCTTGTTGATGGCCCAAATTTGCCCTTCTT
CTTTGGCACTGCTCTTCCAATTGCAGCAGAGGGAGCCTCCTCAGAGGGCCAGTTCCATGG
CGGTTTCAATGGCCGTCTGCAGGTTTGCTGCAACGAAACACACAGACACCCCAAGCTTAA
GGGGGAAGGGAAGGGTTGAAGTCTGCGTGGATCTATCTGATGTTTCTGTTCACTGGATCT
GAAGACATAGAAGCAGAGTGATACTCAACCTTGGGCGCTTGGGTTTTAGTGCTGAAGCCT
GTCTTTCTGCTTCATCAGCTGAGTAATTGTTTTAATCTACTTTTTATGCAATTGTGATTG
TACTGGTGTTTGGTTTTGTGATCTGATAAGCTAAAATTTTTATTTTATCTCCAAGTATAC
ATTTCTTTACTATCAAAGCCTGAATGATGATATTTGCAATTTTTGGCCCCAGAT
>comp1326_c0_seq1 len=2788 path=[1822481:0-2787]
GGGGGCGGCGATCATCTCCTTCCCCATAACGGGGGCACATGGTTAATAACGCCGTCGGAG
GGGCGGTCCCGCCATTGTTTTTGCCAGGTAAAAGTAGAACCCACCCACCCTGTCGCGTAG
CCCTCTTTAGGCCTACCGAGCCAAGGACACACATCTGTAGCGTGCTGTCGGGCACAACTT
CTCATTCATCTCTCTCTCACTGCGGGAAGCCATTACTGCTCTTCCTCCTCCTCCGAACGA
GTTCAAACTTCCACTGAATGGAAGCAGTAAAAAAATAAAAATAAAAACAGAGTCTGGGCT
CGAGCAGTAATTTGGAGAGGGAGGGGTGCTCATTACTCCCTCGTCATCCACATAGAAAGC
AGCGGTGAAATATCCTAAGAGCTGAAAGGAGTCGCTCTCACAACTCAAAAAGACATTGTT
AGAGAGGAGAGGGAATAGAGATAGAAAGAGAAAGGGGGGTACAAACGCAGTAAGTAGCTT
TTGCGAGCAAGTAGAGAGGATCTAGCCGTTATGCGCCTAGCTTAGAGAGAGAGAGAGACA
GGGGTTTCGACTTTCCTGCCATTGTGCGGCCCTCCATTATTATCTCCAAAACAGAGGTTT
GTGCCGGGAAGTGAGAGAAGTTGATTGTAGGCTGTCTGCTTAGTGCGTCTTTGTTGGGAG
ATGGGATGAATTAGCCTCATCTTTAATCAATGACGCCTGTGACGCCGTCCTTCCCCTACT
CTTGTTTCTTTCACCTCCTCCCCCTTCCAGCTAATATGTGATGACTTTTGCGCTGCTTTT
TCTGCCTTAACAGTATCAAAATGGGATCCTGATTTCGTCATCTGTCTTGTGATGGAGCGG
TTGGATGGGCATGGTTTGTCCAAGCGCCACAACTGGTCTTTGCCGGAGCAAGAAGAAAGA
GAGAAAGCACCGTTCTTTTAAGAGCGGGAAGCACGGGAAAGGGAAAAGTAGTGGAGGAGA
AGGAGGGGAAGAAGACGGAGAGTCTGGATCCCAGGAGGAGTTGGCCAGACCAAAGCGAAG
GAGATCGAGGGAGAGACGGAGAAGTTTACCATCACCCGCTGCATCCGTACTTCCCGTGCG
CTTCTTCTTCCTCCGATTATGCGCGGTTGTCGGAGAGCATGGTGGACACGCAGCGGCGGC
CGCGCGCGATGAGGGGCGCCGGAGGGGAGATTGTTGAAGTCCAGGGCGGCGGCCACATTG
TGCGGGCTACGGGGCGAAAGGACCGACACAGCAAGGTATGCACCGCCAAAGGACTAAGAG
ACCGTCGCGTGCGTCTCTCCGCCCACACCGCTATCCAGTTCTATGACGTGCAAGATCGCC
TTGGCCTCGACCGGCCGAGCAAGGCCGTCGACTGGTTGATTAAGAACGCCAAGCCCTCCA
TCGACCAGCTTGCCCAGCTCCCTCCATGGCGTCCTCCAGATCCCTCTCCGCCACACGCGT
CCGCCGGAGACCACGAGGGACAGAAACATCACGTTTCTACTGATATCGGTGACTACGGTG
CTTCTGCTTACGGATATTGCGGTAGTTCGAGCTTGCTCCCTCCTTCGCTCGACTCCGATT
CAATCGCCGACACCATTAAATCATTCTTCCCGAGGACCGCCGCCGTCACCGCCTCTGCAT
CCCCCTCTTATCCCTCGGCCCTCGACCTTCTCTCCCATGGTGGTGGTGGAAGTAGAAGCC
ACGCGGAGGACCTCCGCCTCTCACTCCAGTCCTTCCAGGATCCAATATTTAGTCATCAAC

AGAATCGGGCAGCTCCCGCCCACCAATCCCTTTTTTCCGCTCCCATGAGTTTCGATGCCG
CCGCCGCCTCCACTTGGCCGGAGCCAGGCCAGCGAGCGGCAGCGTGGAATCTTGCCGAAC
CCTCCTCCGCCGAGGCCGGCGGCAGCGGAGGCAGGTACTTGTTTGGCTTATCATCACAAC
TGGTCCAGAACCAGTTTGTCACACAGTGGGGTACCCTTCAGTCCAGTAACCCGCCTTTGA
TTCGTGCCATGGCGGACCCAATCGCTTTATCTTCCGCAGCCGGTCACCAGATGCACCTAA
CAGCTTTTCGGTCGAGCTTAACCTTGCCGGTGCCAGCCTACCAAAACGCCGCCGGCGATG
TGGTTGGCTTCTCTGGGTACCATATGCCAGCACGAATACATGGCGGGGAGGAGCACGATG
GCGCCGTCGGCGACGCGCCTCCCTCTGCCTCCTCTGCTTTGCACCGCTGATGGAAAGTAG
TGAGAAGAGGATCTGAAATAGCTTCAAACGTCTCGTCATCAGATTTTGTCCTGGATTCTT
CGCCGGGCGAAAAGGATCTGCAACTCCGGCAGGCAAAACCAGTTCCTCTGTCTCTCTTTC
CCTTCGGTGTTTTATCTTCTTGTTGGGCTCTCTACAAGCCCTGTATCCGCATTTCTTAAT
TATGTTTTCGCTGTTAACGTTTGATTTATCGTATGTTATTACCGCTGTGCTCCTCTCTCA
TGTCCTCTGTTCCTCCCCCTTCTTCCATTCCAAAGACTTGAACTTAAATTATATATTGTT
AAAGATTGTTTTTTTGCTGGTTTGTTGGTTGGTCTGTGGAAGGGGAGAAATGAGGAAATT
GGAGAAGAAGGATCGATGTATTAGTGGCTGGACACGGACATGAATGAAGCCATGCGTCAC
TTTCATGATGACAGTGAGAAAGCGAGCAGAGCGCCTTGCTGTATAATTGGAAAGAGACTT
GTCCTTTTCCCTTCTTTTGTGACTCTTTTGTTTGCATTTCGAATGGAAAATGGAGATCTG
GGAGATGAAGACGTGGTTTTCTGAAAAA

Supplementary Table S1 The sequence identifier, name or annotation and type of the TCP proteins of *Arabidopsis thaliana*, *Dendrobium hybrid cultivar*, *Oryza sativa*, *Phalaenopsis equestris* and *P. hybrid cultivar* examined.

**Species**	**Identifier**	**Name or annotation**	**Type**
Arabidopsis thaliana	At1g67260	AtTCP1	CYC/TB1
Arabidopsis thaliana	At4g18390	AtTCP2	CIN
Arabidopsis thaliana	At1g53230	AtTCP3	CIN
Arabidopsis thaliana	At3g15030	AtTCP4	CIN
Arabidopsis thaliana	At5g60970	AtTCP5	CIN
Arabidopsis thaliana	At5g41030	AtTCP6	PCF
Arabidopsis thaliana	At5g23280	AtTCP7	PCF
Arabidopsis thaliana	At1g58100	AtTCP8	PCF
Arabidopsis thaliana	At2g45680	AtTCP9	PCF
Arabidopsis thaliana	At2g31070	AtTCP10	CIN
Arabidopsis thaliana	At2g37000	AtTCP11	PCF
Arabidopsis thaliana	At1g68800	AtTCP12	CYC/TB1
Arabidopsis thaliana	At3g02150	AtTCP13	CIN
Arabidopsis thaliana	At3g47620	AtTCP14	PCF
Arabidopsis thaliana	At1g69690	AtTCP15	PCF
Arabidopsis thaliana	At3g45150	AtTCP16	PCF
Arabidopsis thaliana	At5g08070	AtTCP17	CIN
Arabidopsis thaliana	At3g18550	AtTCP18	CYC/TB1
Arabidopsis thaliana	At5g51910	AtTCP19	PCF
Arabidopsis thaliana	At3g27010	AtTCP20	PCF
Arabidopsis thaliana	At5g08330	AtTCP21	PCF
Arabidopsis thaliana	At1g72010	AtTCP22	PCF
Arabidopsis thaliana	At1g35560	AtTCP23	PCF
Arabidopsis thaliana	At1g30210	AtTCP24	CIN
Oryza sativa	Os04g11830	OsPCF1	PCF
Oryza sativa	Os08g43160	OsPCF2	PCF
Oryza sativa	Os11g07460	OsPCF3	PCF
Oryza sativa	Os01g11550	OsPCF5	CIN
Oryza sativa	Os03g57190	OsPCF6	CIN
Oryza sativa	Os01g55100	OsPCF7	CIN
Oryza sativa	Os12g42190	OsPCF8	CIN
Oryza sativa	Os03g49880	OsTB1	CYC/TB1
Oryza sativa	Os01g55750	OsTCP5	CIN
Oryza sativa	Os01g69980	OsTCP6	PCF
Oryza sativa	Os02g42380	OsTCP7	PCF
Oryza sativa	Os02g51280	OsTCP9	PCF

Oryza sativa	Os02g51310	OsTCP10	CIN
Oryza sativa	Os04g44440	OsTCP17	PCF
Oryza sativa	Os05g43760	OsTCP18	CIN
Oryza sativa	Os06g12230	OsTCP19	PCF
Oryza sativa	Os07g05720	OsTCP21	CIN
Oryza sativa	Os08g33530	OsTCP22	CYC/TB1
Oryza sativa	Os09g24480	OsTCP24	CYC/TB1
Oryza sativa	Os09g34950	OsTCP25	PCF
Oryza sativa	Os12g02090	OsTCP27	CIN
Oryza sativa	Os12g07480	OsTCP28	PCF
Dendrobium hybrid cultivar	DQ517495	Dhyb_TCP1	CIN
Phalaenopsis hybrid cultivar	HQ439603	Phyb_TCP1	CIN
Phalaenopsis hybrid cultivar	HQ439604	Phyb_TCP2	CIN
Phalaenopsis hybrid cultivar	HQ439605	Phyb_TCP3	CIN
Phalaenopsis hybrid cultivar	HQ439606	Phyb_TCP4	PCF
Phalaenopsis hybrid cultivar	HQ439607	Phyb_TCP5	CIN
Phalaenopsis equestris	PEQU_03822	TCP7_ARATH Transcription factor TCP7 OS=Arabidopsis thaliana GN=TCP7 PE=2 SV=1	PCF
Phalaenopsis equestris	PEQU_15634	PCF2_ORYSJ Transcription factor PCF2 OS=Oryza sativa subsp. japonica GN=PCF2 PE=1 SV=1	PCF
Phalaenopsis equestris	PEQU_19398	TCP15_ARATH Transcription factor TCP15 OS=Arabidopsis thaliana GN=TCP15 PE=2 SV=1	PCF
Phalaenopsis equestris	PEQU_21454	TCP2_ARATH Transcription factor TCP2 OS=Arabidopsis thaliana GN=TCP2 PE=2 SV=1	CIN
Phalaenopsis equestris	PEQU_09751	PCF2_ORYSJ Transcription factor PCF2 OS=Oryza sativa subsp. japonica GN=PCF2 PE=1 SV=1	PCF
Phalaenopsis equestris	PEQU_14328	TCP4_ARATH Transcription factor TCP4 OS=Arabidopsis thaliana GN=TCP4 PE=2 SV=1	CIN
Phalaenopsis equestris	PEQU_19547	TCP2_ARATH Transcription factor TCP2 OS=Arabidopsis thaliana GN=TCP2 PE=2 SV=1	CIN
Phalaenopsis equestris	PEQU_21260	TCP14_ARATH Transcription factor TCP14 OS=Arabidopsis thaliana GN=TCP14 PE=2 SV=1	PCF
Phalaenopsis equestris	PEQU_25177	TCP15_ARATH Transcription factor TCP15 OS=Arabidopsis thaliana GN=TCP15 PE=2 SV=1	PCF
Phalaenopsis equestris	PEQU_06996	TCP3_ARATH Transcription factor TCP3 OS=Arabidopsis thaliana GN=TCP3 PE=2 SV=1	CIN
Phalaenopsis equestris	PEQU_08153	TCP2_ARATH Transcription factor TCP2 OS=Arabidopsis thaliana GN=TCP2 PE=2 SV=1	CIN
Phalaenopsis equestris	PEQU_40981	TCP23_ARATH Transcription factor TCP23 OS=Arabidopsis thaliana GN=TCP23 PE=1 SV=1	PCF
Phalaenopsis equestris	PEQU_28429	TCP20_ARATH Transcription factor TCP20 OS=Arabidopsis thaliana GN=TCP20 PE=1 SV=1	PCF
Phalaenopsis equestris	PEQU_39718	TCP20_ARATH Transcription factor TCP20 OS=Arabidopsis thaliana GN=TCP20 PE=1 SV=1	PCF
Phalaenopsis equestris	PEQU_06749	TB1_MAIZE Transcription factor TEOSINTE BRANCHED 1 OS=Zea mays GN=TB1 PE=2 SV=2	CYC/TB1
Phalaenopsis equestris	PEQU_06750	TB1_MAIZE Transcription factor TEOSINTE BRANCHED 1 OS=Zea mays GN=TB1 PE=2 SV=2	CYC/TB1
Phalaenopsis equestris	PEQU_11715	TB1_MAIZE Transcription factor TEOSINTE BRANCHED 1 OS=Zea mays GN=TB1 PE=2 SV=2	CYC/TB1

Supplementary Table S2 The nucleotide sequence of the primers used.

**Name**	**Target**	**Forward**	**Reverse**	**Application**
TB1_orchid_TCP	CYC/TB1-like genes	AGRAARGAYMKRCAYARHAAGAT	YTTCTTCTCCAARGTYCTYTCYCT	PCR on genomic DNA
comp8378_c0_seq1	comp_8378	CCAGCTATCTCAGGACGGTT	ATAGCGGCTAGCAGGTTGAG	Real Time PCR
comp8964_c0_seq1	comp_8964	CAGTCTCCGGGAGGTACG	ACAAAGAGGCGAGGAGGTT	Real Time PCR
comp21881_c0_seq1	comp_21881	GGATTCTTGCAGCCCTTAAC	GAGTCACTCGTGCTCATCGT	Real Time PCR
comp16641_c0_seq1	comp_16641	CATATGGGACAGGGAAGAGG	GAGTCGACCCATCAGAATCA	Real Time PCR
comp24776_c0_seq1	comp_24776	GGCTCGAGCTAGGACTTTCA	GTGCTGTTGATGGTGATGCT	Real Time PCR
comp13386_c0_seq1	comp_13386	CCCTTGCAGTTTATGTCGAG	AAGGTTCGAATCAGCAATCC	Real Time PCR
comp12442_c0_seq1	comp_12442	CCGGAACTATACCTGCCATC	GAAGTGGATGATCGGAACCT	Real Time PCR
comp16313_c0_seq1	comp_16313	TTTCCATCATGCAAGACCAT	AGGTGAATTGGACTGAAGGG	Real Time PCR
comp5062_c0_seq1	comp_5062	GTCAGCTCCAGGGATTGACT	AGCAGTGCCAAAGAAGAAGG	Real Time PCR
comp1326_c0_seq1	comp_1326	TGGTCCAGAACCAGTTTGTC	GCTGTTAGGTGCATCTGGTG	Real Time PCR
comp21123_c0_seq1	comp_21123	CCCACAAGCTTCTTCCAGTT	AACAGCATGGCCGTGTAATA	Real Time PCR
TB1_Oita_TCP	OitaTB1	CAAGTTCTTCGATCTCCAGGAT	GACGAGCTCTTTAATGGCTGATT	Real Time PCR
REAL_5.8S_ITA	5.8S	GGATATCTTGGCTCTCGCAT	GATGGTTCACGGGATTCTG	Real Time PCR
Stem-Loop_miR319	miR319		GTCGTATCCAGTGCAGGGTCCGAGGTATTCGCACTGGATACGACAGGGAG	Stem-Loop Real Time PCR
miR319F	miR319	GCGGCGGTTGGACTGAAGGGAG		Stem-Loop Real Time PCR
Stem-Loop_5.8S	5.8S		GTCGTATCCAGTGCAGGGTCCGAGGTATTCGCACTGGATACGACGATTCA	Stem-Loop Real Time PCR
REAL_5.8S_ITA_F	5.8S	GGATATCTTGGCTCTCGCAT		Stem-Loop Real Time PCR
Stem-Loop_Univ_Rev	Universal		GTGCAGGGTCCGAGGT	Stem-Loop Real Time PCR
1326_Rout	comp_1326		GAAGCCAACCACATCGCCGGCGGCG	modified 5' RACE
1326_Rinn	comp_1326		GCTGTTAGGTGCATCTGGTG	modified 5' RACE
5062_Rout	comp_5062		CCTGCAGACGGCCATTGAAACCGCC	modified 5' RACE
5062_Rinn	comp_5062		AGCAGTGCCAAAGAAGAAGG	modified 5' RACE
16313_Rout	comp_16313		CCGGCTGCATGTATGCCTCATTACAG	modified 5' RACE
16313_Rinn	comp_16313		CTGGTTCTCCAGCGAGCTTCCCGCTG	modified 5' RACE
